# Utilization of Galectins by Pathogens for Infection

**DOI:** 10.3389/fimmu.2020.01877

**Published:** 2020-08-19

**Authors:** Diyoly Ayona, Pierre-Edouard Fournier, Bernard Henrissat, Benoit Desnues

**Affiliations:** ^1^Aix Marseille Univ, IRD, APHM, MEPHI, IHU-Méditerranée Infection, Marseille, France; ^2^Aix-Marseille Univ, IRD, APHM, VITROME, IHU-Méditerranée Infection, Marseille, France; ^3^Architecture et Fonction des Macromolécules Biologiques, CNRS, Aix-Marseille University, Marseille, France; ^4^USC1408 Architecture et Fonction des Macromolécules Biologiques, Institut National de la Recherche Agronomique, Marseille, France; ^5^Department of Biological Sciences, King Abdulaziz University, Jeddah, Saudi Arabia

**Keywords:** galectin, infection, immune responses, bacteria, virus

## Abstract

Galectins are glycan-binding proteins which are expressed by many different cell types and secreted extracellularly. These molecules are well-known regulators of immune responses and involved in a broad range of cellular and pathophysiological functions. During infections, host galectins can either avoid or facilitate infections by interacting with host cells- and/or pathogen-derived glycoconjugates and less commonly, with proteins. Some pathogens also express self-produced galectins to interfere with host immune responses. This review summarizes pathogens which take advantage of host- or pathogen-produced galectins to establish the infection.

## Introduction

**Figure 1:**
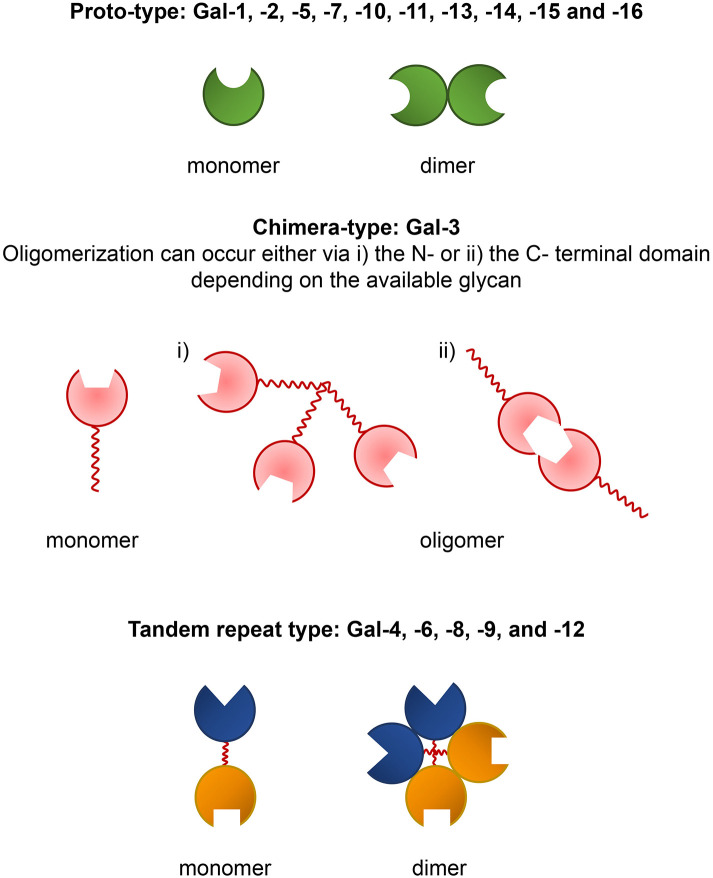
Prototype, chimera, and tandem repeat-type galectins and their modes of self-association.

Galectins are glycan-binding proteins characterized by evolutionary conserved carbohydrate recognition domains (CRDs) with affinity for β-galactose-containing glycoconjugates. The first mammalian galectin, Gal-1, was discovered in the late 1970's, and today a total of 16 different mammalian galectins have been identified. These lectins are categorized into three major groups, called “prototypal,” “tandem repeat,” and “chimeric” based on their structural differences. The prototypal galectins have one CRD and the members are Gal-1, -2, -5, -7, -10, -11, -13, -14, -15 (1) and -16 (2). Tandem repeat galectins, Gal-4, -6, -8, -9, and -12 comprise two distinct CRDs which are connected by a short linker peptide (1). These galectins are sometimes called “heterodimeric” because of their distinct CRDs (3). Gal-3 is the only known chimeric galectin in vertebrates. It consists of one CRD bearing an extended N-terminal domain with unusual proline-, glycine-, and tyrosine-rich repeats (1). Prototypical galectins are monomers which preferentially form non-covalent homodimers in solution resulting in a monomer-dimer equilibrium (Figure 1). The dimeric form of prototypal galectins is critical for galectin-glycan binding and cell signaling (4, 5). Tandem repeat galectins are usually reported to be monomeric in solution, but they may form homodimers or multimers (Figure 1), as described for Gal-9 (6, 7), which could be more effective in forming a network with cell surface glycoproteins (8, 9). Gal-3 predominantly exists as a monomer in solution (10), but can self-associate via its N-terminal (11) or C-terminal domains (12) (Figure 1). It has been shown that Gal-3 self-association is dependent on the ligand (13) and the concentration of monomeric Gal-3. Oligomerization of Gal-3 can result in high molecular weight aggregates (14). It has been suggested that Gal-3 could form pentamers based on stoichiometry in precipitates with some synthetic divalent glycans (15). However, the data in this paper are also compatible with other models, better supported by additional data [summarized in Lepur et al. (13) and Johannes et al. (16)]. A key feature is that a bound Gal-3 molecule enhances binding of additional galectin-3 molecules. In addition to glycans, Gal-3 has also the ability to interact with distinct proteins such as Alix (17) or Bcl-2 via its N-terminal domain (18).

In humans, a total of 13 genes encoding galectins have been identified, including two genes for Gal-9 (1, 2). The primary carbohydrate unit recognized by all galectins is Gal-β(1,4)-GlcNAc (*N*-acetyllactosamine, LacNAc) (8, 19). Hence, LacNAc-containing glycolipids like gangliosides and glycoproteins, such as mucins, laminin, lysosome-associated membrane proteins and fibronectins can serve as endogenous ligands for vertebrate galectins (20). The galectin binding affinity for a ligand varies for each galectin and thus, each galectin has a specific glycan affinity spectrum (21). For example, Gal-1 has high affinity for complex type *N*-glycans (K_d_ <8 μM) and its affinity increases with the N-glycan branching number (21). Gal-3 has a particular preference for blood group A (K_d_ ~3.8 μM) and B (K_d_ ~1.6 μM) saccharides. In addition, Gal-3, Gal-8, and Gal-9 have high affinity (K_d_ <2 μM) toward repeats of LacNAc. Interestingly, the binding affinities of the N- and C-terminal domains of tandem repeat Gal-8 can vary, even for the same ligand (21). Recent studies have highlighted that sialylation (and particularly α2,6-sialylation) of the terminal galactose residues has an inhibitory effect on galectin-glycan interactions [see (22) for a review].

Galectins have a wide tissue distribution (23). However, the abundance of individual galectins varies with the tissue and the cell type. Moreover, the expression of some galectins is highly modulated by external stimulations, including microbial infection (16, 24). Galectins can be found inside (in the cytosol, nucleus, mitochondria or associated with the plasma membranes) or outside the cells. Sometimes, depending on location, the same galectin may have a different impact on a certain process (25). Thus, galectin secretion is tightly regulated. Although galectins are devoid of secretion signal peptide sequences, they are secreted outside the cells by non-classical pathways where they interact with extracellular matrix and cell surface glycans (16, 24). Although galectins can sometimes function in a glycan-independent manner, their primary function is mediated through carbohydrate recognition (26).

Galectins are involved in a broad spectrum of cellular functions, including cell adhesion, migration, apoptosis, autophagy, signaling, proliferation, and growth as well as in different non-inflammatory and inflammatory diseases like asthma, obesity, diabetes, fibrosis, cardiovascular disease, and cancers (24, 27–29). Galectins are preferentially expressed in myeloid than in lymphoid cells, and myeloid cells appear to be the main targets for galectin-mediated regulation of immune responses (30).

**Figure 2:**
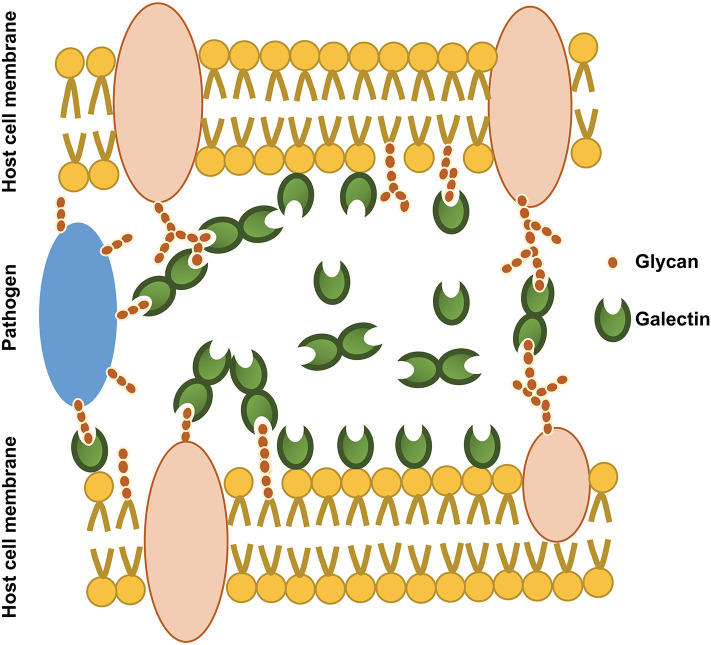
An illustration of the involvement of secreted galectins in host-host glycan and host-microbial glycan interactions. Secreted galectins can exist as free soluble molecules in the extracellular space or associated with cell membrane and extracellular matrix. Secreted galectins may bind host glycans (cell surface glycans), foreign glycans (pathogen surface glycans) and even bridge host glycans and pathogen glycans.

Galectin targets are not limited to endogenous cell glycans. They can also bind the surface glycans of pathogens (Figure 2), acting as pattern recognition receptors (PRRs) (28). Numerous studies have shown the impact of infection on host galectin expression, emphasizing their role in host defense. Galectins can negatively interfere with mechanisms crucial for pathogen adherence and cell entry. For example, Gal-1 inhibits the adsorption and internalization of Dengue virus (DENV-1) by directly interacting with the virus (31). Galectin binding on the pathogen surface can also exert direct and indirect antimicrobial effects. The prime example for this is Gal-3, which is also well-known for its fungicidal activity. Acting as a PRR, Gal-3 can kill *Candida* spp. bearing β-1,2-linked oligomannosides (32). Similarly, Gal-3 inhibits the growth of *Streptococcus pneumoniae* by augmenting neutrophil function against bacteria (33). Moreover, both Gal-4 and -8 tandem repeat galectins can specifically recognize blood group antigen B on some bacteria to eradicate them (34). Gal-3 not only acts as a PRR but also acts as an opsonin. During *E. coli* infection, Gal-3 binds lipopolysaccharide (LPS) and thus, increases the phagocytic killing of bacteria (35). Intracellular Gal-3 participates to bacterial killing by directing the delivery of antimicrobial guanylate binding proteins to intracellular pathogen-containing vacuoles, such as those containing *Legionella pneumophila* or *Yersinia pseudotuberculosis*. The infected vacuoles are detected by sensing of bacterial secretion systems inserted in the membranes (36). Gal-8 and -9 can also detect vesicle-damaging pathogens via exposed host glycans from ruptured membranes, and Gal-8 was shown to activate antibacterial autophagy (37). Finally, galectins also regulate immune responses by modulating cytokine production (38), and by regulating the activation and recruitment of immune cells (33, 39). Therefore, galectins play a vital role against infections [see (40) for a review].

In spite of the involvement of galectins against infection, some pathogens have evolved strategies to use and exploit galectins to facilitate their adherence and entry into cells and to subvert host immune responses in order to favor invasion (28, 40). This review discusses how host-galectins are directly or indirectly hijacked by some pathogens and how pathogen self-produced galectins can act in favor of infection.

## Viral Infections

Viral capsids and envelope proteins are important determinants of virus-host interaction and invasion (41). They can sometimes be *N*- or *O*- glycosylated by the host glycosylation machineries (41–43). In addition, glycosylation can also be affected by mutations of the glycosylation motifs of the viral proteins (44). Glycosylated viral proteins play a crucial role by actively participating to viral entry and by interfering with host immune responses. Viral glycoproteins are therefore important therapeutic and diagnostic targets (40).

### Human Immunodeficiency Virus Type 1 (HIV-1)

Galectins interact with HIV-1 at different stages of the viral cycle (45). At the cell entry stage, Gal-1 interaction with the Gp120 envelope protein bridges Gp120 with its T cell receptor, CD4 (46, 47). *N*-linked glycans from CXCR4 and CCR5 co-receptors of Gp120 are composed of 70% of oligo-mannose while the remainder is represented by bi-, tri-, and tetra-antennary complex glycans bearing two to six β-galactose residues. Affinity chromatography has shown that complex *N*-glycans released from Gp120 have relatively similar affinities for Gal-1 and Gal-3, but only Gal-1 can bind *N*-glycans attached to Gp120 (46). Interestingly, Gal-1 and Gal-3 retain the same affinity for Gp120 even when disulfide bridges are reduced, suggesting that the binding difference between Gal-1 and Gal-3 is independent of the structure of complex glycans. Therefore, this binding difference has been assumed to arise from the spatial organization of the complex glycans on Gp120 (46). It has also been found that Gal-1 binds CD4 in a β-galactose-dependent manner. CD4 affinity chromatography has shown that the presence of Gal-1 increases the retention of Gp120 on the CD4 column and thus that Gal-1 stabilizes the Gp120-CD4 interaction (46). Accordingly, it has been proposed that oligo-mannose and complex glycans form homogeneous clusters on Gp120 surface and that these clusters may be more abundant near the CD4+ binding site of Gp120, possibly facilitating Gal-1-mediated Gp120-CD4 interactions (46). In line with these findings, *in vitro* cell infection assays demonstrated that the addition of recombinant Gal-1 enhances the infection (46, 48) while mutations affecting complex viral glycans result in reduced infectivity (49).

Gal-3 interaction with HIV-1 was also shown to be exploited by the virus at later stages of the viral life cycle. The association between the p6 domain of the viral Gag protein (a viral protein involved in virion release) and ALG-2-interacting protein X (Alix, a cell cytoskeletal protein involved in endocytosis) is crucial for viral budding. Intracellular Gal-3 interacts with the proline-rich regions of Alix via its N-terminal domain (50). Accordingly, co-immunoprecipitation experiments and cell infection assays using cells deficient for Gal-3 or Alix have revealed that Gal-3-mediated Alix interaction promotes viral budding by stabilizing the Gag-p6-Alix complex (50, 51).

Gal-9 has also been shown to favor HIV-1 infection by inducing Th1 cell apoptosis and Th2 cell migration, thus decreasing Th1/Th2 cell ratio (52). The T cell immunoglobulin mucin domain 3 (Tim-3) and non-sialylated *N*-glycans are essential requirements for Gal-9 mediated T cell apoptosis. Gal-9 specifically targets Th1 cells over Th2 cells, because Th2 cells lack Tim-3 and express α-2,6 sialylated *N*-glycans (53, 54). Instead of Tim-3, Gal-9 binds *O*-glycans on the T cell receptor protein disulfide isomerase (PDI) of Th2 cells. PDI, which is more abundant on Th2 than Th1 cells (52), has been shown to catalytically rearrange disulfide bonds of the viral envelope glycoproteins and this is crucial for effective membrane fusion and cell entry (55). Immunoprecipitation and T cell migration assays have revealed that Gal-9-PDI interaction induces Th2 cell migration by increasing the abundance as well as the retention time of PDI on Th2 cell surface while regulating β3 integrin activation (52). Accordingly, it has been shown that exogenous addition of Gal-9 increases the viral entry in a PDI-dependent manner, since PDI inhibitors reverted the Gal-9-mediated viral entry *in vitro*. In summary, the Th2 microenvironment resulting from Gal-9 upregulation is exploited by HIV-1 to promote infection (52).

### Human T Cell Leukemia Virus Type 1 (HTLV-1)

The formation of virological synapses following host cell-to-cell contact is essential for the transmission of HTLV-1. These virological synapses result from the interaction between infected and non-infected cells. HTLV-1-infected cells secrete Gal-1 at higher levels than uninfected cells. This was found to be the result of activation of Gal-1 promoter by viral protein Tax (56). *In vitro* cell infection assays have shown that virions infection can be increased by adding Gal-1 and can be inhibited by lactose, which interferes the galectin interaction with host cell/viral β-galactose residues (56). Quantitative T cell fusion assays have shown that Gal-1 enhances HTLV-1-mediated T cell fusion of infected cells with uninfected cells. Gal-1 is thus believed to increase the cytoplasmic exchange of the virus through gp46-dependent cell fusion events between infected and non-infected cells (56). In addition to virological synapse formation, HTLV-1 cell-to-cell transmission can occur by other means. Indeed, HTLV-1 budding virions have also been found embedded in adhesive, virus-induced extracellular matrices at the surface of infected cells. These extracellular matrices promote rapid adherence of infected cells to other lymphocytes. Interestingly, the matrices contain extracellular components as well as cellular proteins, including Gal-3 but not Gal-1 (57, 58). The precise role of Gal-3 in this biofilm pathway remains to be elucidated.

### Herpes Simplex Virus (HSV-1)

HSV-1 uses different strategies to escape T cell-mediated viral clearance, such as the increase of T cell apoptosis, the inhibition of T cell activation as well as the secretion of pro-inflammatory cytokine (59–62). Gal-1 can also mediate apoptosis of Th1, Th17 and cytotoxic T cells by interacting with LacNAc-containing *N*- or *O*-glycans harbored by receptors such as CD7, CD43, and CD45. As described earlier, Th2 cells are not affected due to differentially sialylated surface glycoproteins. It has been shown *in vitro* that HSV-1 induces expression and secretion of Gal-1 and thereby promotes cell apoptosis which can be inhibited by Gal-1 inhibitors (60). Hence, HSV-1 appears to use Gal-1 to promote T cell apoptosis and to reduce T cell-mediated virus clearance (60, 63). However, in a murine model of herpetic keratitis, Rajasagi et al. have shown that recombinant Gal-1 treatment suppressed stromal lesions and decreased disease severity (64). Therefore, the exact role of Gal-1 during HSV-1 infection remains to be clarified.

Gal-3 is found on the glycocalyx of the apical cell membrane of corneal keratinocytes. Affinity assays have shown that Gal-3 interacts with HSV-1 and transmembrane mucins. Intriguingly, knockdown of Gal-3 prevents HSV-1 infection of human corneal keratinocytes. Contradictorily, binding of Gal-3 to transmembrane mucins on apical membranes appeared to reduce HSV-1, as indicated by reduced HSV-1 infectivity *in vitro* with the addition of transmembrane mucins. Therefore, Gal-3 seems to play a dual role acting as an entry mediator of HSV-1 during herpetic keratitis (65).

HSV-1 latency is mainly maintained by CD8+ T cells accumulating in the trigeminal ganglion (TG). Reddy et al. have evaluated the role of Gal-9 during HSV-1 latency *in vivo*. They observed an increase in Gal-9 expression of TG as well as Tim-3 expression of resident CD8+ T cells, 3–10 days after infection. Interestingly, CD8+ T cells isolated from TG of infected Gal-9-deficient mice had a higher effector functionality than those isolated from wild-type mice, as indicated by their relatively high production of IFN-γ or TNF-α cytokines. Moreover, addition of recombinant Gal-9 to TG cell cultures in the latent phase resulted in apoptosis of the majority of CD8+ T cells. Therefore, it has been concluded that the Gal-9/Tim-3 pathway not only induces CD8+ T cell apoptosis but also impairs the CD8+ T cells effector functionality (66). HSV-1 reactivation from latency can occur when the number or effector function of CD8+ T cells in TG is altered (67, 68). Accordingly, it has been shown that infection of Gal-9-deficient TG cells results in a delay and a reduction of viral reactivation from latency. Hence, Gal-9 appears to favor HSV-1 infection by facilitating viral reactivation from latency under the influence of the host homeostatic mechanisms (66).

### Influenza Virus

Influenza virus harbors an envelope densely covered with galactose, oligomannose, and sialic acid residues. The two major envelope glycoproteins are hemagglutinin (HA) and neuraminidase (NA). Their glycosylation type is either complex or oligomannose and varies depending on the viral subtypes and strains (69, 70). HA attaches viruses to host cell surface by binding to sialylated receptors and facilitates penetration by favoring fusion of viral to endosomal membranes. NA removes sialic acid from viral and host cell glycoproteins to trigger virion mobility for cell entry or release. Therefore, the balance between HA and NA activity is crucial for efficient viral replication (71). It has been found that pre-incubation of Madin-Darby Canine Kidney (MDCK) cells with Gal-1 and Gal-8 promotes virus binding, but the infectivity varies depending on strain type and galectin dose (69). Yang et al. have shown that in the presence of Gal-1, the viral load is significantly reduced after 24 h compared to the control without Gal-1. Moreover, they demonstrated that Gal-1 interaction with influenza virus glycans limits the infection *in vivo*, by reducing viral load, inflammation, and cell apoptosis in the lung. It is hypothesized that Gal-1 may limit the binding of HA to sialylated receptors by promoting Gal-1 mediated cross-linking of host and viral cell surface glycans (70). Accordingly, human Gal-1 functional variants that display higher Gal-1 expression levels show protection against influenza infection (72).

### Epstein-Barr Virus (EBV)

Patients with EBV-mediated lymphoproliferative disorders show a characteristic T cell dysfunction. After the primary infection, EBV can persist in a latent state in resting and proliferating cells. EBV has three latency programs with distinct patterns of latent gene expression. The most immunogenic form of EBV latency, called type 3, is maintained in long-lived memory B lymphocytes and type 3 latency is characterized by the expression of several EBV nuclear antigens, two small RNAs and two latent membrane proteins called LMP1 and LMP2 (73). It has been demonstrated *in vitro* that LMP1 and LMP2 induce the activation of Gal-1 transcription via AP-1 and PI3K/Akt cell signaling pathways. Furthermore, it was found that addition of recombinant Gal-1 induces apoptosis of EBV specific CD8+ T cells and that apoptosis can be abrogated in the presence of Gal-1-neutralizing antibodies. Therefore, overexpression of Gal-1 upon EBV infection is thought to be a strategy of EBV-induced immune evasion via Gal-1-mediated Th1 cell apoptosis. This may explain why the EBV infection develops within a Th2-predominant microenvironment (63).

Another *in vitro* study on EBV-associated nasopharyngeal carcinomas has suggested a role for Gal-9 in favor of infection. EBV-associated nasopharyngeal carcinoma produce Gal-9 containing exosomes (74). These exosomes protect Gal-9 from proteolytic cleavage and preserve its Tim-3 binding capacity. Accordingly, it has been shown that these exosomes induce the EBV-specific CD4+ T cell apoptosis via the Tim-3/Gal9 pathway and that apoptosis can be inhibited by neutralizing antibodies directed against the Tim-3-binding domain of Gal-9. Such antibodies could be a promising route to maintain CD4+ T cell survival (75).

### Enterovirus 71 (EV71)

The non-enveloped EV71 has four capsid proteins (VP1 to VP4) and four other non-structural proteins. Immunofluorescence and immunoprecipitation studies revealed that host cellular Gal-1 interacts with the virus in infected cells, particularly with VP1 and VP3. Interestingly, the released virions are associated with Gal-1, forming a Gal-1/virion complex. Thus, Gal-1 not only interacts with EV71 within the host cells but also with released virions (76). Cell infectivity assays have shown that the binding and infectivity of Gal-1-free virions is lower than that of the Gal-1/virion complexes and this reduced infectivity can be recovered by adding recombinant Gal-1. In addition, mice infected with Gal-1-free virions display a high survival rate with mild neuropathological symptoms while mice infected by Gal-1/virion complexes have severe symptoms and a low survival rate. Additionally, cell infectivity assays performed under high and low temperatures revealed that EV71/Gal-1 complexes are more resistant than Gal-1-free virions against thermal and environmental stress. Therefore, during infection Gal-1 contributes to binding, infectivity, pathogenicity, and stability of EV71. This may explain the high level of Gal-1 in the serum from children with severe EV71 infection (76).

Gal-3 may also be involved in EV71 infection as an inducer of virion release. Gal-3 deficient mice have reduced intracellular viral load and viral release compared to wild-type mice 24 h after infection. Cell viability assays on EV71-infected wild-type and Gal-3-deficient cells in the presence of apoptosis inhibitors have suggested that Gal-3 induces the release of virions following EV71-mediated apoptosis. However, Gal-3 impact on EV71 may also depend on Gal-3 genetic variants. Indeed, cells expressing the non-synonymous Gal-3 genetic variant called rs4644 (one of the major Gal-3 single nucleotide polymorphism with minor allele frequency) produce lower virus amounts compared to cells expressing the wild-type allele. It would be interesting to address the impact of Gal-3 variants on EV71 disease severity (77).

### Nipah Virus

Nipah virus preferentially infects microvascular endothelial cells, and cell entry is mediated by two envelope glycoproteins called NiV-F and NiV-G. The NiV-G glycoprotein binds primarily to host cell entry receptor ephrinB2 or alternatively ephrinB3 while NiV-F mediates the fusion of bound virus with host cells (75). The *N*-glycans of NiV-F are important modulators of cell fusion. NiV-F and NiV-G are also expressed at the surface of infected host cells and this can trigger host cell-to-cell fusion resulting in endothelial syncytia formation and endothelial disruption (78). In response to viral infection, endothelial cells produce inflammatory mediators, including galectins (79).

Cell infectivity assays have shown that addition of recombinant Gal-1 enhances cell attachment and subsequent cell entry of the virus by bridging viral and host *N*-glycans. Removal of viral/host *N*-glycans reduces the viral adherence to host cells. Particularly, mutants lacking the *N*-glycans on NiV-F3 (F3 is a specific *N*-glycosylated site on NiV-F) have a significant reduction of Gal-1-mediated attachment. It has also been shown that Gal-1 addition before infection increases syncytia formation, but Gal-1 addition after the infection reduces the progeny virus production and syncytia formation (79). Mutants affecting NiV-F3 *N*-glycans were found to be resistant against Gal-1 mediated promotion or inhibition of syncytia formation. The increased syncytia formation by Gal-1 has been suggested to be a consequence of amplified viral envelope production by initially infected cells, which would increase the availability of viral *N*-glycans at the surface of infected cells (79). In a previous study, Garner et al. have proposed three different ways for Gal-1 to inhibit syncytia formation: (i) by delaying the maturation of NiV-F, (ii) by reducing the lateral mobility of NiV-F on the plasma membrane, and (iii) by abrogating the conformational change of NiV-F required to stimulate the fusion (78). Therefore, the effect of Gal-1 appears to depend on the timing of Gal-1 exposure and on the positioning of *N*-glycans on the NiV-F3 site (79). According to the model proposed by Garner et al., these F3 *N*-glycans are positioned above the fusion peptide and proximal to the target cell membrane. The distance between the two CRDs of dimeric Gal-1 is too short to link the F3 *N*-glycans of two F promoters on the same NiV-F spike. Therefore, dimeric Gal-1 binds with *N*-glycans of NiV-F3 from different F spikes resulting in a network built by multiple low-affinity glycan-galectin interactions and this network blocks the triggering of fusion peptide (79). Based on this model, it has been suggested that Gal-1 interaction with NiV-F on infected cells enhances network formation and, thereby, blocks syncytia formation. In contrary, when Gal-1 interacts with NiV-F on virions, the network formation become less efficient, perhaps due to the tightly packed envelope glycoproteins and consequently Gal-1 bridges the virus to host cells. Binding to host cell receptors triggers the fusion of F spikes when the virus particles are not bound to Gal-1. In summary, Gal-1 acts in favor of the infection at the initial stage of virus attachment while at later stage it may reduce the viral progeny and syncytia formation (79).

## Bacterial Infections

Numerous studies have reported the ability of galectins to directly interact with bacterial surface glycans. Gram-negative bacterial lipopolysaccharides (LPS) are major pathogen associated molecular patterns and are well-known ligands for Gal-3 [see (40) for a review]. LPS have three major components: the hydrophobic lipid A which anchors LPS to the outer membrane, the oligosaccharide part comprising the inner and outer cores, and the O-antigen that is connected to lipid A by the core oligosaccharides (80). Depending on the bacteria and their particular LPS structure, Gal-3 mainly binds to β-galactoside glycans from the outer core or the O-antigen. Of note, the LPS from *Salmonella minnesota* which lacks β-galactose residue, is bound by Gal-3 through its lipid A (81). In contrast, Gram-positive bacterial cell walls contain peptidoglycan, which is covered with teichoic and lipoteichoic acids, which lack β-galactose residues. It was shown that galectins can in this case bind hydrophobic ligands from such Gram-positive bacteria (82, 83).

### *Salmonella* spp.

Gal-3 interaction with LPS can act as a negative regulator of LPS-induced endotoxic shock (84) [see (85) for a review]. This has also been observed with *S. minnesota* LPS. Exposure of Gal-3-deficient mice to *S. minnesota* LPS induces higher expression of IL-12, IL-6, TNFα, NO, and H_2_O_2_ compared to wild-type mice and this over-production of inflammatory cytokines can be reduced when LPS is pre-incubated with Gal-3. Gal-3-LPS interaction also prevents the downstream signaling of TLR4, as indicated by the reduced phosphorylation of JNK1/2, ERK1/2, p38, and NF-κBp65. Thus, Gal-3-deficient mice are more susceptible to LPS-induced endotoxic shock than wild-type mice (86). However, exposure to LPS enhances the serum Gal-3 level in wild-type mice, suggesting a possible involvement of Gal-3 in a feedback regulation of LPS-mediated inflammatory response. Indeed, the absence of Gal-3 skews the immune response toward a Th1 response, which is associated with high production of bactericidal reactive oxygen species. Hence, it is thought that Gal-3 favors the early stages of *Salmonella* infection by acting as a negative regulator of endotoxic shock (86).

### Listeria monocytogenes

A study done to evaluate the role of Gal-3 during *Shigella, Salmonella*, and *Listeria* infections has shown that Gal-3 is rapidly recruited at the site of the lysed vacuoles where Gal-3 interacts with β-galactose-bearing glycoconjugates. Thus, Gal-3 has been proposed to be a marker of vacuole lysis by pathogens (87). Weng et al. have reported that Gal-3 interferes with the bacterial clearance by reducing NO production by macrophages during *L. monocytogenes* infection (88). This study has also clarified the importance of Gal-3 accumulation at *L. monocytogenes*-containing phagosomes (89). The bacteria have a poorer level of intracellular replication and survival in Gal-3 deficient cells compared to wild-type cells, and the intracellular bacterial count in Gal-3 deficient cells can be further reduced by adding autophagy inhibitors. Hence, Gal-3 interference with the autophagy process is exploited to favor infection (89).

Gal-8 is known to promote antibacterial autophagy by binding glycans exposed on damaged vacuolar membranes (37, 89). It has been shown that Gal-8/Gal-3-double deficient cells display a significantly lower bacterial replication than Gal-8-deficient cells. This result emphasizes the ability of Gal-3 to suppress autophagy, independently of Gal-8 (89). However, when complex *N*-glycans are depleted from these two types of cells, the bacterial replication is significantly reduced only in Gal-8-deficient cells compared to cells without *N*-glycan depletion. Thus, *N*-glycans are essential for autophagy suppression mediated by Gal-3 and, instead of being a competitor of Gal-8 for *N*-glycans, Gal-3 appears to actively participate to the down regulation of antibacterial autophagy (89).

### Neisseria meningitidis

Immunohistochemical analysis of tissues infected by *N. meningitidis* have shown that the bacterial colonies in infected tissues co-localize with Gal-3 and also elevate the expression of Gal-3. The interaction of bacteria with Gal-3 appears to be primarily mediated by the bacterial LPS, as demonstrated by the decreased Gal-3 binding of bacteria in LPS mutants or in presence of lactose. Meningococcal strains that harbor LPS immunotypes with terminal LacNAc residues interact well with Gal-3. Addition of recombinant Gal-3 facilitates the adhesion of bacteria to macrophages and monocytes, but not to epithelial cells. However, Gal-3 deficiency does not affect meningococcal phagocytosis, suggesting that only soluble Gal-3 is involved in the enhancement of the infection depending on the cell type (82).

Based on the fact that neither lactose nor mutations of LPS are able to completely inhibit Gal-3 binding to bacteria, other authors have suggested that Gal-3 may bind the bacteria independently of the LPS (82, 90). This was supported by the successful binding of lactose-liganded recombinant Gal-3 to bacteria. However, lactose-liganded Gal-3 binding was reduced when bacterial mutants lacking pilins (PilE or PilQ) were used. Experimental evidence and molecular modeling have suggested that Gal-3 may be involved in a complex tripartite interaction connecting the host cell non-integrin laminin receptor, the meningococcal secretin PilQ and the major pilin PilE. It has also been shown that the reduced bacterial invasion observed in Gal-3-deficient cells could be restored by Gal-3 transfection, suggesting a role for intracellular Gal-3 during infection. Further work is required to clarify the actual role of the LPS-independent Gal-3-binding in bacterial invasion (90).

### Porphyromonas gingivalis

During *P. gingivalis* infection, *in vitro* association and invasion assays have shown that addition of Gal-1, but not of Gal-3, enhances bacterial adhesion and invasion in gingival epithelial cells. Nevertheless, *in vitro* Gal-1 or Gal-3 deficiency had no effect on bacterial adhesion and invasion. Therefore, enhancement of *P. gingivalis* infection by Gal-1 appears to be mediated by soluble Gal-1, but not cellular Gal-1 (91). *P. gingivalis* fimbriae is known to promote adhesion and invasion by interacting with β1-integrin (92). Interestingly, Gal-1 has the ability to interact with both bacterial LPS and β1-integrin. Therefore, during *P. gingivalis* infection soluble Gal-1 may enhance infection by linking microbial glycans to the oligosaccharide chains of β1-integrin (91, 93).

### Group A Streptococcus (GAS)

GAS are well-known extracellular pathogens, yet they are able to persist within non-immune as well as immune cells (94). They can trigger their internalization into non-phagocytic cells such as epithelial and endothelial cells (95, 96). Utilizing cells for cytosolic replication enables the bacteria to avoid antibiotics and immune-mediated killing (96, 97). Unlike endothelial cells, GAS are efficiently killed in epithelial cells within lysosome-fused autophagosomes. In endothelial cells, the autophagy-mediated killing is insufficient to completely eliminate the bacteria. This allows the bacteria to persist and replicate. The difference in the autophagy-mediated killing by these two cell types resides on the differential expression of Gal-3 and Gal-8. Immunofluorescence imaging has shown that Gal-3 and Gal-8 are recruited toward GAS without changing the intracellular expression of Gal-3 and Gal-8-encoding genes. Interestingly, endothelial cells recruit more Gal-3 while epithelial cells recruit more Gal-8 (97). Ubiquitination is a post-translational modification required for autophagy. During anti-bacterial autophagy, parkin is an important protein which directs the ubiquitin recruitment to the surface of the pathogen and of pathogen-containing vesicles. Experiments conducted with infected Gal-3-deficient cells revealed that the lack of Gal-3 enhances the recruitment of ubiquitin, parkin and Gal-8 as well as increases the interaction of parkin with Gal-8 (97). Further analyses revealed that Gal-3 attenuates the recruitment of Gal-8, parkin and ubiquitin to GAS. Consequently, Gal-3-deficient mice have less severe GAS-induced skin damage and reduced bacterial replication than the wild-type mice. This outlines the importance of the Gal-8/parkin interaction for ubiquitin recruitment and the ability of Gal-3 to inhibit Gal-8/Parkin-mediated ubiquitination. Thus, it appears that Gal-3 attenuates the ubiquitination of GAS and GAS-containing vacuoles in endothelial cells where Gal-3 is more abundant, thereby interfering with autophagy-mediated killing of bacteria (97). Of note, the ability of Gal-3 to block the recruitment of parkin and ubiquitin may also explain the Gal-3-mediated autophagy suppression observed in *L. monocytogenes* (88, 97).

### Helicobacter pylori

*H. pylori* harbors an O-antigen side chain composed of a glucosylated LacNAc backbone (98). Gal-3 has been found to mediate *H. pylori* O-antigen adhesion to gastric epithelial cells. The interaction between Gal-3 and O-antigen occurs in a carbohydrate dependent manner as indicated by the reduced adherence of O-antigen side chain mutants or by addition of lactose. Accordingly, it has been demonstrated that *in vitro* Gal-3 transfection into Gal-3-deficient cells restores bacterial adhesion. The bacteria also induce the expression and secretion of Gal-3 upon infection (99, 100). Interestingly, mutants lacking cytotoxin antigen A or inhibition of MAPK cell signaling reduce Gal-3 expression (99, 100). However, Subhash et al. have reported that extracellular Gal-3 decreases bacterial adherence to the cells and reduces apoptosis of infected cells (100). In line with this finding, *in vivo* experiments have also shown that *H. pylori* can successfully colonize the gastric glands of both wild-type and Gal-3-deficient mice models, but with a higher bacterial load in Gal-3-deficient mice (101). *H. pylori* regulates multiple signaling pathways including MAPK by CagA-mediated mechanisms to modulate cell proliferation and apoptosis (102). Because Gal-3 plays many roles in cancer (103), understanding the interplay between Gal-3, CagA and development of *H. pylori* mediated gastric carcinogenesis is important to develop proper remedies (104).

Moreover, *H. pylori* induces autophagy in gastric epithelial cells by expressing vacuolating cytotoxin A (VacA) to limit toxin-induced cell damage. However, extended exposure to VacA suppresses autophagy to prevent maturation of autolysosome to ensure intracellular survival and persistence of the bacteria. Therefore, the dynamic balance between formation and suppression of autophagosomes is critical for bacterial survival (105). In gastric epithelial cells, *H. pylori* infection induces lysosomal damage. Gal-8 recognizes exposed vacuolar host *O*-glycans in damaged lysosomes. Accumulation of Gal-8 triggers the formation of intracellular Gal-8 aggregates, which colocalize with damaged lysosomes as well as with autophagosomes (106). It has been shown that Gal-8 deficiency reduces the activation of bacteria-mediated autophagy, possibly via the resulting dysfunction of ubiquitination (97, 106). Therefore, Gal-8 also seems to be important for *H. pylori* survival by participating to autophagy vesicle formation (105, 106).

### Pseudomonas aeruginosa

Studies of mice corneas infected with *P. aeruginosa* have revealed that bacterial infection down-regulates expression of Gal-3 and up-regulates that of Gal-7, Gal-8, and Gal-9. The functions of these galectins during infection are not clearly defined (107). Nevertheless, Gal-3 has been shown to interact with bacterial LPS, particularly with the outer core region. In addition, antibodies directed against bacterial LPS or Gal-3 significantly reduce bacterial binding to mouse cornea. Hence, Gal-3 interaction with LPS appears to be crucial for bacterial interaction with the corneal epithelium (108).

### Klebsiella pneumoniae

During *K. pneumoniae* infection, IL-17A plays a crucial role in host defense against the infection (109). IL-17A is produced by Th17 cells which are also susceptible to Gal-9/Tim-3-mediated cell apoptosis. Accordingly, it has been shown that injection of Gal-9 to *K. pneumoniae-*infected mice reduces the cytokine secretion by Th1 and Th17 cells and also inhibits the up-regulation of G-CSF (granulocyte-colony stimulating factor) and MIP-2 (Macrophage Inflammatory Protein 2), which are crucial for neutrophil differentiation (110). Thus, injection of Gal-9 to infected mice reduces bacterial clearance, resulting in a lower survival rate than the control group without Gal-9 treatment. Collectively, these findings suggest that Gal-9/Tim-3-mediated apoptosis of Th1 and Th17 cells act as a negative regulator of host defense mechanism against *K. pneumoniae* (110).

### Proteus mirabilis

Among different types of fimbriae expressed by *P. mirabilis*, the non-agglutinating fimbriae which are important for adherence to uroepithelial cells (111), have been found to interact with Gal-3 (112). Indeed, it was shown that bacteria binding to MDCK cells could be prevented by the presence of anti-Gal-3 antibodies (112). Given the ability of non-agglutinating fimbriae to interact with glycolipids, including asialo-GM1, -GM2 and lactosyl ceramide *in vitro* (113), it is possible that Gal-3 facilitates such interactions and thereby mediates bacterial adherence to host cell receptors (112).

### Yersinia enterocolitica

*Y. enterocolitica* infection induces Gal-1 expression in the Peyer's patches and the spleen. The modulation of Gal-1 is dependent on two of *Yersinia* outer proteins (Yops), YopP, or YopH, since YopP and YopH mutants are associated with reduced Gal-1 expression *in vivo* (114). During *Y. enterocolitica* infection, YopP suppresses pro-inflammatory signaling pathways while YopH inhibits phagocytosis by dephosphorylating adaptors of cell signaling pathways (115). Gal-1 up-regulation is also dependent on MAPK cell signaling, since the induction of Gal-1 can be abolished by disturbing the MAPK cell signaling cascade. Interestingly, *in vivo* Gal-1 deficiency was shown to be associated with a high survival rate through enhanced proinflammatory response as revealed by increased expression of TNFα, NO, NF-κB activation, IFN-γ, and IL-17. Thus, there is an interplay between the Yops, Gal-1 and MAPK cell signaling cascade that controls innate immunity resulting in mitigated mucosal proinflammatory responses (114).

### Chlamydia trachomatis

The high mannose *N*-glycans carried by high molecular weight (MW) *C. trachomatis* glycoproteins are well-known for their role in bacterial attachment and infectivity, as indicated by the reduced infectivity of *N*-glycanase-treated bacteria (116). A recent study has highlighted that LacNAc-containing low MW glycoproteins (<55 kDa) are also important determinants of infection by their ability to facilitate Gal-1 mediated host/bacteria interactions. Lectin blotting has shown that Gal-1 preferentially binds these low MW glycoproteins, but not the high MW glycoproteins due to their α2,6 sialylation. Western blots and immunofluorescence imaging of infected cells show that Gal-1 expression is upregulated upon infection, and its subcellular distribution is changed (117). *In vivo* and *in vitro* assays have revealed that the addition of recombinant Gal-1 enhances bacterial adherence as well as invasion. Interestingly, the complex *N*-glycans on the host cells enhance infectivity by interacting with Gal-1. Indeed, it was shown that the absence of complex β1,6 branched *N*-glycans on host cell surface was sufficient to decrease infectivity. These results suggest that homodimeric Gal-1 bridges the bacterial low MW glycoproteins to the host cell complex *N*-glycans and thereby facilitates bacterial adherence and internalization. Additionally, it was found that Gal-1 colocalizes with both PDGFRβ and β_1_/α_V_β_3_ integrins and inhibition of these two integrins significantly reduces the bacterial infection promoted by Gal-1. Thus, it appears that integrins are also involved in the Gal-1-mediated enhancement of infection (117).

## Parasitic Infection

A parasite life cycle typically involves different stages with distinct composition, exposure and availability of the glycoconjugates. Such changes allow the parasite to adapt and survive in the specific environments relevant to each stage. Some parasites harness galectin binding to their glycoconjugates to facilitate infection in different ways at different stages of their life cycle (118).

### Trypanosoma cruzi

*T. cruzi*, the causative agent of Chagas disease, expresses surface *O*- and *N*-glycoconjugates with terminal β-galactose residues capped with sialic acid (119). *T. cruzi* is present in different forms in its insect vector and in mammals. In mammals, trypomastigotes can infect different cell types and once inside the cells, they differentiate into amastigotes. By contrast, inside the insect gut, trypomastigotes differentiate into epimastigotes. Later, epimastigotes differentiate into metacyclic trypomastigotes which initiate the infection in mammals (120).

Galectin-binding assays have shown that *T. cruzi* has affinity for Gal-1, -3, -4, -7, and -8. Remarkably, galectins preferentially bind the infective forms, i.e., amastigotes and trypomastigotes, which are more efficiently recognized by host cells than the non-infective epimastigotes (118). *In vitro* exogeneous addition of Gal-3 enhances trypanosome binding to host cells (121) and binding to laminin-coated wells (122). In line with these observations, it has been shown that Gal-3 inhibitors interfere with *T. cruzi* invasion *in vitro* (123). Gal-7, the main galectin expressed on skin and stratified epithelia, binds epimastigotes and increases the infection *in vitro*. In addition, it has been found that trypomastigotes have a lower binding efficiency to Gal-8-deficient cells. Collectively, these observations suggest that galectin-mediated interactions promote the adhesion of the infective form to their target cells (118). At the same time, Gal-1 has been shown to prevent *T. cruzi* infection as well as further damages on cardiac cells (124). The persistence of *T. cruzi* in cardiac tissues causes chronic myocarditis and remodeling of the cardiac matrix. The parasitic burden and intensity of Chagas heart disease vary with different *T. cruzi* genotypes. Evaluation of Chagas heart disease in mice caused by different *T. cruzi* genotypes has shown that the expression of pro-collagen-1 and Gal-3 are correlated to fibrosis, parasitemia, and parasitic burden. Therefore, Gal-3 is thought to be associated to the remodeling of the cardiac extracellular matrix and thus to the severity of the Chagas heart disease (125). Using Gal-3-deficient mice, it was found that Gal-3 controls parasitic burden and tissue damage differently in acute and chronic phases (126). Even though its role in *T. cruzi* infection is still far from understood, Gal-3 is considered to favor Chagas disease particularly at the early stages (125–127). Collectively, the different observations suggest that galectins play a multi-faceted role during *T. cruzi* infection.

### Trichomonas vaginalis

*T. vaginalis* is an obligatory extracellular pathogen whose adherence to cervicovaginal epithelial cells is critical to its survival (128). *T. vaginalis* expresses surface lipophosphoglycans (LPGs) which are composed of a ceramide phosphoinositol glycan core containing abundant LacNAc repeats (129). *In vitro*, LPGs mediate *T. vaginalis* cytotoxicity and adherence. However, the adherence of *T. vaginalis* is not mediated solely by LPGs and it also involves galectins (130). Indeed, galectin-binding assays have shown that *T. vaginalis* binds to Gal-1 and the binding can be drastically decreased in the presence of lactose or with LPG mutants. Moreover, *in vitro* exogenously addition of Gal-1 increases parasite adherence while Gal-1-deficient cells are characterized by decreased parasite binding (131).

Cell infection assays in the presence of different Gal-1 or Gal-3 concentrations, have shown that the parasite regulates Gal-1 level differently based on the soluble Gal-1 concentration, but predominantly down regulates Gal-3. Moreover, elevated Gal-1 was found to be associated with the suppression of chemokines important to eliminate extracellular protozoa (e.g., IL-8, MIP-3α) while Gal-3 appears to play an opposite role (132). Interestingly, Gal-1 also stimulates chemokine expression, which in turn enhances Gal-3 production. The absence of Gal-1 results in a higher proinflammatory response, while the lack of Gal-3 suppresses the inflammation. Therefore, it seems that the parasite takes advantage of Gal-1 and Gal-3 to modulate host immune responses (132).

### *Plasmodium* spp.

During experimental cerebral malaria caused by *P. berghei* ANKA, moribund mice display a significantly higher Gal-3 expression in brain tissue samples than non-moribund mice. Moreover, Gal-3 deficient mice had a 50% reduction in development of cerebral malaria compared to wild-type. However, adherence assays have shown that this reduction was independent of Gal-3 binding to *P. berghei*. Therefore, Gal-3 appears to favor *Plasmodium* infection by other means than promoting adherence (133). It was also found that Gal-3 displays a species-specific impact on parasitaemia and antibody-mediated responses. Indeed, Gal-3 deficiency results a significant reduction and minor increase, respectively, for *P. yoelii* and *P. chabaudi*. In the case of *P. yoelii*, the reduction of parasitemia in Gal-3-deficient mice was found to be associated to an increase in anti-IgG against *P. yoelii* merozoite surface protein 119 (134). Thus, Gal-3 may favor infection by modulating the production of cytophilic IgG in a species-specific manner.

### Leishmania donovani

During visceral leishmaniasis, the number of T cells increases in liver and spleen. As a result, Gal-1 gene expression increases in liver and spleen upon infection. Under these circumstances, regulatory T cells (Tr1) express more Gal-1 than Th1 cells, but the latter have a higher cell count. Interestingly, it has been found that during the acute phase of infection, Gal-1-deficient mice have a significantly decreased parasitic load and an increased count of pro-inflammatory cytokine-producing CD4+ T cells in the liver compared to infected wild-type mice. Therefore, Gal-1 appears to restrict CD4+ T cell response during acute hepatic *L. donovani* infection and thereby may limit anti-parasitic immune response by T cell apoptosis (135).

## Fungal Infections

Galectins can recognize the surface glycans of fungi (40). In particular Gal-3 has been shown to exert both fungicidal and fungistatic effects (40, 136). However, some fungi have evolved strategies to utilize Gal-3 to favor infection (136). Interestingly, Gal-3 can associate with dectin-1, a C-type lectin on host cells that mediates direct fungal uptake by recognizing fungal cell wall β-1,3-glucan (40).

### *Candida* spp.

Both macrophages and neutrophils have the ability to phagocyte *Candida* spp. (137, 138) and Gal-3 is involved in phagocytosis of *Candida* spp. by neutrophils. The efficacy of phagocytosis by neutrophils varies with the fungal species and the biological phases. For example, *C. albicans* hyphae and *C. parapsilosis* yeast are more effectively phagocytized by neutrophils than *C. albicans* yeast. This observation was further confirmed by the inability of neutrophils to phagocyte *C. albicans* hyphae and *C. parapsilosis*, but not *C. albicans* yeast, in the presence of Gal-3 blocking agents (138). However, challenging the protective role of Gal-3 against *C. albicans* yeast infection, *in vivo* Gal-3 deficiency was shown to enhance the fungicidal activity against systemic *C. albicans* infection by increasing the production of reactive oxygen species (ROS). Mechanistically, it was shown that cytosolic Gal-3 interferes with complement receptor-3 (CR3) downstream signaling. Evaluation of ROS production by Gal-3-deficient neutrophils and CR3-deficient neutrophils revealed that ROS production in CR3-deficient neutrophils is low while it is high in Gal-3-deficient neutrophils. Other experiments have confirmed that Gal-3 directly interacts with spleen tyrosine kinase protein (Syk) upon *Candida* infection and Syk inhibitors reduce ROS production by neutrophils. Thus, Gal-3 favors *C. albicans* by negatively regulating CR3 downstream Syk-mediated ROS production (139). However, Linden et al. showed that Gal-3 deficiency reduces the survival of mice infected with *C. albicans* but not *C. parapsilosis* (140). These divergent observations may be the result of different inoculum size, experimental settings and other *in vivo* factors (139, 140).

### Histoplasma capsulatum

*H. capsulatum* is an opportunistic pathogen, which resides in macrophages phagolysosomes (141). Both Th1 and Th17 responses play a crucial role in clearing this fungal infection (142). Comparative analysis of cytokine production by bone marrow-derived dendritic cells from wild-type and Gal-3-deficient mice revealed that the lack of Gal-3 enhances the expression of TGF-β1 and IL-23, suggesting that Gal-3 downregulates Th17 response. Accordingly, it has been shown that Gal-3-deficient mice infected with *H. capsulatum* have a reduced fungal burden compared to wild-type mice (17). Moreover, Gal-3-deficient mice produce more Th17 cytokines but less Th1 cytokines than wild-type mice. Upon infection, IL-17A production by both Gal-3-deficient and wild-type neutrophils is higher than that of CD4+ T cells and is increased by the addition of recombinant IL-23. Therefore, it appears that high levels of IL-23 in Gal-3-deficient mice trigger the production of IL-17 by neutrophils and thereby enhance Th17 responses. In line with this, it was shown that Gal-3 expression by dendritic cells inhibits the production of IL-23/IL17-A in response to *H. capsulatum* infection and thereby reduces fungal clearance (17).

## Galectins From The Pathogen or From The Pathogen's Vector

Mammals are not the sole producers of galectins. Indeed, galectins and galectin-like molecules have been found in chicken, insects, fungi, nematodes, amphibians, eel, sea urchins, fish, and sponges (143–145). While some pathogens exploit the galectins from their vector, some use self-produced galectins to favor of the infection (146, 147).

### *Leishmania* spp.

*Leishmania* species are responsible of a wide spectrum of diseases in humans. They are transmitted via insect vectors such as the sand fly *Phlebotomus papatasi*. Attachment of *Leishmania* promastigotes to the mid gut of the sand fly is essential for the completion of the parasitic cycle and this binding is mediated by a tandem repeat galectin of *P. papatasi* called PpGalec. PpGalec is expressed in the midgut and interacts with lipophosphoglycan, the main surface glycoconjugate of *Leishmania* promastigotes (146, 148).

### Haemonchus contortus

*H. contortus*, a gastrointestinal parasitic nematode of small ruminants, produces a tandem repeat galectin called Hco-gal. The N- and C-terminal CRDs of Hco-gal bind to goat peripheral blood mononuclear cells. The transmembrane receptors TMEM63A and TMEM147 were found to be receptors, respectively, for the N- and C-terminal CRDs of Hco-gal (149). The Hco-gal C-terminal CRD is more effective than the N-terminal one in inducing cell apoptosis, inhibiting cell proliferation and suppressing IFN-γ transcription, but the N-terminal CRD is more potent in inhibiting NO production. This functional difference between N- and C- terminal CRDs could be a result of their receptor binding specificities. Collectively it is thought that Hco-gal contributes to the parasitic immune evasion by altering cellular activities including cytokine production (149).

### Trichinella spiralis

*T. spiralis* is a zoonotic nematode that produces a tandem repeat type galectin, Tsgal, which can be found at all developmental stages of the parasite. Tsgal has an hemagglutination function and can also bind intestinal epithelial cells at sites that are located on the membrane and in the cytoplasm to promote larval invasion of intestinal epithelium at the early stage of infection (150).

### Dirofilaria immitis

*D. immitis*, is a parasitic nemetode commonly known as heartworm. It expresses a galectin that acts as a physiological plasminogen receptor which alters the fibrinolytic balance. Thus, this galectin promotes plasmin production to prevent clot formation in its surrounding environment (151). Additionally, interfering the host fibrinolytic system assists the long term persistence of the parasite resulting in chronic infection (152).

### Coronaviruses

Some coronaviruses S glycoprotein spike proteins have N-terminal domains (NTD) that show similarities to the structural fold of galectins. Carbohydrate or protein moieties are usually the binding receptors of such NTDs (153, 154). For example, NTDs of human coronavirus OC43 and bovine coronavirus have sugar-binding sites structurally similar to human galectins and recognize cell surface 9-O-acetyl-sialic acid for attachment (154). Instead, NTDs from murine coronavirus binds to carcinoembryonic antigen-related cell adhesion molecules which are important for cell entry as a surface attachment factor. It has been suggested that these galectin-like domains may be important for attachment to host cells and also for zoonotic transmission (153, 154).

## Conclusions and Future Prospect

Galectins are ubiquitous molecules which have a broad spectrum of binding partners and functions. This functional diversity may explain why some pathogens have evolved strategies to exploit galectins to favor infection. Among the different mammalian galectins, Gal-1, -3, and -9 have a significant role in the modulation of the immune response. Their abundance and secretion by target and infected cells probably explains why these three galectins appear more involved in infections than other galectins (28, 40) (Table 1 and Supplementary Table 1). The promotion of viral infections appears restricted to these three galectins, most probably because of the reduced diversity of viral glycoconjugates compared to that of other pathogens. Interestingly, galectins can exert positive or negative effects on the same infection. The relative abundance of these three galectins is also crucial for the establishment of infection. Generally, Gal-1 and Gal-3 increase adherence as well as immune evasion while the contribution of Gal-9 is mostly restricted to immune evasion. Gal-3 appears more often involved in different infections than the other two galectins, perhaps due to its unique structure. Independent of carbohydrate recognition, the N-terminal extension allows Gal-3 to interact with different cellular proteins such as ESCRT-associated protein Alix. Gal-3 can also oligomerize via its N-terminal as well as C-terminal extensions. In addition, because Gal-3 primarily interacts with internal LacNAc motifs, α-2,6 sialylation of terminal glycans does not abolish binding by Gal-3 (22, 155).

**Table 1:** Host galectin mediated interactions that favor infection; a detailed table is available as supplementary.

**Pathogen**	**Host cell type(s) involved in galectin targeting**	**Galectin(s) favoring pathogen infection**	**References**
HIV-1	T cells	Gal-1	(46, 48)
		Gal-3	(50)
		Gal-9	(53)
HTLV-1	T cells	Gal-1	(56)
		Gal-3	(57)
HSV-1	T cells lacking sialylated cell surface glycoproteins	Gal-1	(60)
	Corneal keratinocytes	Gal-3	(65)
	CD8+ T cell	Gal-9	(66)
Influenza	Alveolar epithelial cells	Gal-1 and -8	(69)
EBV	CD8+ T cells	Gal-1	(63)
	Th1 CD4+ cells	Gal-9	(75)
EV71	Targets cells in different tissues	Gal-1	(76)
		Gal-3	(77)
Nipah	Endothelial cells	Gal-1	(79)
*Salmonella* spp.	Macrophages	Gal-3	(86)
*L. monocytogenes*	Macrophages	Gal-3	(88, 89)
*N. meningitidis*	Phagocytic cells: macrophages, monocytes	Gal-3	(82, 90)
*P. gingivalis*	Gingival epithelial cells	Gal-1	(91)
Group A *Streptococcus*	Endothelial cells	Gal-3	(97)
*H. pylori*	Gastric epithelial cells	Gal-3	(99)
		Gal-8	(106)
*P. aeruginosa*	Corneal epithelial cells	Gal-3	(108)
*K. pneumoniae*	Th1 and Th17 cells	Gal-9	(110)
*P. mirabilis*	Uroepithelial cells	Gal-3	(112)
*Y. enterocolitica*	Cells in spleen and Peyer's patches	Gal-3	(114)
*C. trachomatis*	Cervical epithelial cells	Gal-1	(117)
*T. cruzi*	Different cells vulnerable for infection	Gal-3	(121, 122)
		Gal-7 and -8	(118)
*T. vaginalis*	Vaginal epithelial cells	Gal-1	(132)
*Plasmodium* spp.	Red blood cells	Gal-3	(133, 134)
*L. donovani*	CD4+ T cells	Gal-3	(135)
*C. albicans*	Neutrophils	Gal-3	(139)
*H. capsulatum*	Dendritic cells	Gal-3	(17)

Gal-3 is associated with a high proinflammatory cytokine production (156–158) and leukocyte recruitment (159) and thus is believed to have a predominant proinflammatory function. Additionally, Gal-3 suppresses Th17 response (e.g., *H. capsulatum*), NO production (e.g., *C. albicans*), and autophagy (e.g., Group A *Streptococcus*). Hence, Gal-3 impact on Th1, Th2, and Th17 immune response balance is a critical determinant of infection progression. However, the impact of Gal-3 as a pro-inflammation modulator can vary based on the involvement of T cell apoptosis mediated by Gal-1 and Tim-3/Gal-9. Because sialylated glycans protect Th2 cells from galectin-mediated apoptosis, it would be interesting to assess how sialylation of glycans from the pathogen (e.g., *T. cruzi*) may be involved in distorting the Th1/Th2 balance via galectin-pathogen glycan-mediated interaction.

Through different mechanisms, the same galectin can exert both positive and negative effects on the infection by the same pathogen. This differential regulation is sometimes driven by the phase of the infection or the parasitic stage (e.g., cell entry, cell fusion, chronic and acute infection) as observed during the infections by Nipah virus and *T. cruzi*, respectively. The ability of a galectin to form oligomers, its secretion and localization at the site of infection, its polymorphism, and the genus/species of the pathogen are other factors which can also favor infection.

In summary, a deep understanding of the details of galectin-pathogen interactions is required to better combat pathogens and to avoid treatments which may turn protective galectin-mediated mechanisms into promotion of infection. Given their multiple roles in infectious diseases, galectins have emerged as a modern drug target against a broad range of infections. While galectin-mediated therapeutics are currently limited, many galectin-targeting drugs are under development (160).

## Author Contributions

DA analyzed all relevant literature and drafted the manuscript. P-EF, BH, and BD critically revised the manuscript. All authors have given their approval for this version to be submitted.

## Conflict of Interest

The authors declare that the research was conducted in the absence of any commercial or financial relationships that could be construed as a potential conflict of interest.

## References

[1] Cummings RD Liu F-T Vasta GR Galectins. In: Varki A Cummings RD Esko JD Stanley P Hart GW Aebi M editors. Essentials of Glycobiology. New York, NY: Cold Spring Harbor Laboratory Press (2015). p. 469–80.

[2] Than NG Romero R Goodman M Weckle A Xing J Dong Z. A primate subfamily of galectins expressed at the maternal–fetal interface that promote immune cell death. Proc Natl Acad Sci USA. (2009) 106:9731–6. 10.1073/pnas.0903568106 19497882 PMC2689813

[3] Ludwig A-K Michalak M Shilova N André S Kaltner H Bovin NV. Studying the structural significance of galectin design by playing a modular puzzle: homodimer generation from human tandem-repeat-type (heterodimeric) galectin-8 by domain shuffling. Molecules. (2017) 22:1572. 10.3390/molecules22091572 28925965 PMC6151538

[4] Giudicelli V Lutomski D Lévi-Strauss M Bladier D Joubert-Caron R Caron M. Is human galectin-1 activity modulated by monomer/dimer equilibrium? Glycobiology. (1997) 7:viii–x. 10.1093/glycob/7.3.323-a 9147038

[5] Levroney EL Aguilar HC Fulcher JA Kohatsu L Pace KE Pang M. Novel innate immune functions for galectin-1: galectin-1 inhibits cell fusion by Nipah virus envelope glycoproteins and augments dendritic cell secretion of proinflammatory cytokines. J Immunol. (2005) 175:413–20. 10.4049/jimmunol.175.1.413 15972675 PMC4428613

[6] Miyanishi N Nishi N Abe H Kashio Y Shinonaga R Nakakita S. Carbohydrate-recognition domains of galectin-9 are involved in intermolecular interaction with galectin-9 itself and other members of the galectin family. Glycobiology. (2007) 17:423–32. 10.1093/glycob/cwm001 17223646

[7] Nagae M Nishi N Murata T Usui T Nakamura T Wakatsuki S. Crystal structure of the galectin-9 N-terminal carbohydrate recognition domain from *Mus musculus* reveals the basic mechanism of carbohydrate recognition. J Biol Chem. (2006) 281:35884–93. 10.1074/jbc.M606648200 16990264

[8] Dings RPM Miller MC Griffin RJ Mayo KH. Galectins as molecular targets for therapeutic intervention. Int J Mol Sci. (2018) 19:905. 10.3390/ijms19030905 29562695 PMC5877766

[9] Earl LA Bi S Baum LG. Galectin multimerization and lattice formation are regulated by linker region structure. Glycobiology. (2011) 21:6–12. 10.1093/glycob/cwq144 20864568 PMC2998985

[10] Morris S Ahmad N André S Kaltner H Gabius H-J Brenowitz M. Quaternary solution structures of galectins-1, -3, and -7. Glycobiology. (2004) 14:293–300. 10.1093/glycob/cwh029 14693909

[11] Massa SM Cooper DN Leffler H Barondes SH. L-29, an endogenous lectin, binds to glycoconjugate ligands with positive cooperativity. Biochemistry. (1993) 32:260–7. 10.1021/bi00052a033 8418845

[12] Yang R-Y Hill PN Hsu DK Liu F-T. Role of the carboxyl-terminal lectin domain in self-association of galectin-3 . Biochemistry. (1998) 37:4086–92. 10.1021/bi971409c 9521730

[13] Lepur A Salomonsson E Nilsson UJ Leffler H. Ligand induced galectin-3 protein self-association. J Biol Chem. (2012) 287:21751–6. 10.1074/jbc.C112.358002 22549776 PMC3381137

[14] Hsu DK Zuberi RI Liu FT. Biochemical and biophysical characterization of human recombinant IgE-binding protein, an S-type animal lectin. J Biol Chem. (1992) 267:14167–74. 1629216

[15] Ahmad N Gabius H-J André S Kaltner H Sabesan S Roy R. Galectin-3 precipitates as a pentamer with synthetic multivalent carbohydrates and forms heterogeneous cross-linked complexes. J Boil Chem. (2004) 279:10841–7. 10.1074/jbc.M312834200 14672941

[16] Johannes L Jacob R Leffler H. Galectins at a glance. J Cell Sci. (2018) 131:jcs208884. 10.1242/jcs.208884 29717004

[17] Wu S-Y Yu J-S Liu F-T Miaw S-C Wu-Hsieh BA. Galectin-3 negatively regulates dendritic cell production of IL-23/IL-17–axis cytokines in infection by *Histoplasma capsulatum*. J Immunol. (2013) 190:3427–37. 10.4049/jimmunol.1202122 23455499

[18] Akahani S Nangia-Makker P Inohara H Kim HR Raz A. Galectin-3: a novel antiapoptotic molecule with a functional BH1 (NWGR) domain of Bcl-2 family. Cancer Res. (1997) 57:5272–6. 9393748

[19] Henrick K Bawumia S Barboni EA Mehul B Hughes RC. Evidence for subsites in the galectins involved in sugar binding at the nonreducing end of the central galactose of oligosaccharide ligands: sequence analysis, homology modeling and mutagenesis studies of hamster galectin-3. Glycobiology. (1998) 8:45–57. 10.1093/glycob/8.1.45 9451013

[20] Vasta GR Ahmed H Nita-Lazar M Banerjee A Pasek M Shridhar S. Galectins as self/non-self recognition receptors in innate and adaptive immunity: an unresolved paradox. Front Immunol. (2012) 3:199. 10.3389/fimmu.2012.00199 22811679 PMC3396283

[21] Hirabayashi J Hashidate T Arata Y Nishi N Nakamura T Hirashima M. Oligosaccharide specificity of galectins: a search by frontal affinity chromatography. Biochim Biophys Acta. (2002) 1572:232–54. 10.1016/S0304-4165(02)00311-2 12223272

[22] Zhuo Y Bellis SL. Emerging role of α2, 6-sialic acid as a negative regulator of galectin binding and function. J Biol Chem. (2011) 286:5935–41. 10.1074/jbc.R110.191429 21173156 PMC3057866

[23] Nio-Kobayashi J. Tissue- and cell-specific localization of galectins, β-galactose-binding animal lectins, and their potential functions in health and disease. Anat Sci Int. (2017) 92:25–36. 10.1007/s12565-016-0366-6 27590897

[24] Leffler H Galectins structure and function—a synopsis. In: Crocker PR editor. Mammalian Carbohydrate Recognition Systems. Heidelberg: Springer (2001). p. 57–83. 10.1007/978-3-540-46410-5_4 11190679

[25] Popa SJ Stewart SE Moreau K. Unconventional secretion of annexins and galectins. Semin Cell Dev Biol. (2018) 83:42–50. 10.1016/j.semcdb.2018.02.022 29501720 PMC6565930

[26] Kamili NA Arthur CM Gerner-Smidt C Tafesse E Blenda A Dias-Baruffi M. Key regulators of galectin–glycan interactions. Proteomics. (2016) 16:3111–25. 10.1002/pmic.201600116 27582340 PMC5832938

[27] Brinchmann MF Patel DM Iversen MH. The role of galectins as modulators of metabolism and inflammation. Mediators Inflamm. (2018) 2018:9186940. 10.1155/2018/9186940 29950926 PMC5987346

[28] Vasta GR. Galectins as pattern recognition receptors: structure, function, and evolution. Adv. Exp Med Biol. (2012) 946:21–36. 10.1007/978-1-4614-0106-3_2 21948360 PMC3429938

[29] Yang R-Y Rabinovich GA Liu F-T. Galectins: structure, function and therapeutic potential. Expert Rev Mol Med. (2008) 10:e17. 10.1017/S1462399408000719 18549522

[30] Liu F-T. Regulatory roles of galectins in the immune response. Int Arch Allergy Immunol. (2005) 136:385–400. 10.1159/000084545 15775687

[31] Toledo KA Fermino ML Andrade Cdel C Riul TB Alves RT. Galectin-1 exerts inhibitory effects during DENV-1 infection. PLoS ONE. (2014) 9:e112474. 10.1371/journal.pone.0112474 25392933 PMC4231055

[32] Kohatsu L Hsu DK Jegalian AG Liu F-T Baum LG. Galectin-3 induces death of *Candida* species expressing specific β-1, 2-linked Mannans. J Immunol. (2006) 177:4718–26. 10.4049/jimmunol.177.7.4718 16982911

[33] Farnworth SL Henderson NC Mackinnon AC Atkinson KM Wilkinson T Dhaliwal K. Galectin-3 reduces the severity of pneumococcal pneumonia by augmenting neutrophil function. Am J Pathol. (2008) 172:395–405. 10.2353/ajpath.2008.070870 18202191 PMC2312371

[34] Stowell SR Arthur CM Dias-Baruffi M Rodrigues LC Gourdine J-P Heimburg-Molinaro J. Innate immune lectins kill bacteria expressing blood group antigen. Nat Med. (2010) 16:295–301. 10.1038/nm.2103 20154696 PMC2853181

[35] Cockram TOJ Puigdellívol M Brown GC. Calreticulin and galectin-3 opsonise bacteria for phagocytosis by microglia. Front Immunol. (2019) 10:2647. 10.3389/fimmu.2019.02647 31781126 PMC6861381

[36] Feeley EM Pilla-Moffett DM Zwack EE Piro AS Finethy R Kolb JP. Galectin-3 directs antimicrobial guanylate binding proteins to vacuoles furnished with bacterial secretion systems. Proc Natl Acad Sci USA. (2017) 114:E1698–706. 10.1073/pnas.1615771114 28193861 PMC5338555

[37] Thurston TLM Wandel MP von N Foeglein Á Randow F. Galectin-8 targets damaged vesicles for autophagy to defend cells against bacterial invasion. Nature. (2012) 482:414–8. 10.1038/nature10744 22246324 PMC3343631

[38] Gordon-Alonso M Bruger AM van der Bruggen P. Extracellular galectins as controllers of cytokines in hematological cancer. Blood. (2018) 132:484–91. 10.1182/blood-2018-04-846014 29875102 PMC6073326

[39] Sato S Ouellet N Pelletier I Simard M Rancourt A Bergeron MG. Role of galectin-3 as an adhesion molecule for neutrophil extravasation during streptococcal pneumonia. J Immunol. (2002) 168:1813–22. 10.4049/jimmunol.168.4.1813 11823514

[40] Vasta GR. Roles of galectins in infection. Nat Rev Microbiol. (2009) 7:424–38. 10.1038/nrmicro2146 19444247 PMC3759161

[41] Lucas W Viral Capsids and Envelopes: Structure and Function. Chichester: John Wiley & Sons Ltd; ELS; American Cancer Society (2010).

[42] Aloor A Zhang J Gashash E Parameswaran A Chrzanowski M Ma C. Site-specific N-glycosylation on the AAV8 capsid protein. Viruses. (2018) 10:644. 10.3390/v10110644 30453606 PMC6266768

[43] Murray S Nilsson CL Hare JT Emmett MR Korostelev A Ongley H. Characterization of the capsid protein glycosylation of adeno-associated virus type 2 by high-resolution mass spectrometry. J virol. (2006) 80:6171–6. 10.1128/JVI.02417-05 16731956 PMC1472596

[44] Wang I-N Li Y Que Q Bhattacharya M Lane LC Chaney WG. Evidence for virus-encoded glycosylation specificity. Proc Natl Acad Sci USA. (1993) 90:3840–4. 10.1073/pnas.90.9.3840 7683409 PMC46401

[45] Sato S Ouellet M St-Pierre C Tremblay MJ. Glycans, galectins, and HIV-1 infection. Ann NY Acad Sci. (2012) 1253:133–48. 10.1111/j.1749-6632.2012.06475.x 22524424

[46] St-Pierre C Manya H Ouellet M Clark GF Endo T Tremblay MJ. Host-soluble galectin-1 promotes HIV-1 replication through a direct interaction with glycans of viral gp120 and host CD4. J Virol. (2011) 85:11742–51. 10.1128/JVI.05351-11 21880749 PMC3209312

[47] Yoon V Fridkis-Hareli M Munisamy S Lee J Anastasiades D Stevceva L. The GP120 molecule of HIV-1 and its interaction with T cells. Curr Med Chem. (2010) 17:741–9. 10.2174/092986710790514499 20088758

[48] Mercier S St-Pierre C Pelletier I Ouellet M Tremblay MJ Sato S. Galectin-1 promotes HIV-1 infectivity in macrophages through stabilization of viral adsorption. Virology. (2008) 371:121–9. 10.1016/j.virol.2007.09.034 18028978

[49] Shen R Raska M Bimczok D Novak J Smith PD. HIV-1 envelope glycan moieties modulate HIV-1 transmission. J virol. (2014) 88:14258–67. 10.1128/JVI.02164-14 25275130 PMC4249159

[50] Wang S-F Tsao C-H Lin Y-T Hsu DK Chiang M-L Lo C-H. Galectin-3 promotes HIV-1 budding via association with Alix and Gag p6. Glycobiology. (2014) 24:1022–35. 10.1093/glycob/cwu064 24996823 PMC4181451

[51] Chen H-Y Wang S-F Hsu DKH Chen Y-MA Liu F-T Galectin-3 translocates to virological synapse and promotes HIV-1 transfer (VIR1P. 1000). Am Assoc Immnol. (2014) 192 (1 Supp. 74.14).

[52] Bi S Hong PW Lee B Baum LG. Galectin-9 binding to cell surface protein disulfide isomerase regulates the redox environment to enhance T cell migration and HIV entry. Proc Natl Acad Sci USA. (2011) 108:10650–5. 10.1073/pnas.1017954108 21670307 PMC3127870

[53] Bi S Earl LA Jacobs L Baum LG. Structural features of galectin-9 and galectin-1 that determine distinct T cell death pathways. J Biol Chem. (2008) 283:12248–58. 10.1074/jbc.M800523200 18258591 PMC2431002

[54] Zhu C Anderson AC Schubart A Xiong H Imitola J Khoury SJ. The Tim-3 ligand galectin-9 negatively regulates T helper type 1 immunity. Nat Immunol. (2005) 6:1245–52. 10.1038/ni1271 16286920

[55] Fenouillet E Barbouche R Courageot J Miquelis R. The catalytic activity of protein disulfide isomerase is involved in human immunodeficiency virus envelope-mediated membrane fusion after CD4 cell binding. J Infect Dis. (2001) 183:744–52. 10.1086/318823 11181151

[56] Gauthier S Pelletier I Ouellet M Vargas A Tremblay MJ Sato S. Induction of galectin-1 expression by HTLV-I Tax and its impact on HTLV-I infectivity. Retrovirology. (2008) 5:105. 10.1186/1742-4690-5-105 19032754 PMC2613925

[57] Pais-Correia A-M Sachse M Guadagnini S Robbiati V Lasserre R Gessain A. Biofilm-like extracellular viral assemblies mediate HTLV-1 cell-to-cell transmission at virological synapses. Nat med. (2010) 16:83–9. 10.1038/nm.2065 20023636

[58] Hsu DK Hammes SR Kuwabara I Greene WC Liu FT. Human T lymphotropic virus-I infection of human T lymphocytes induces expression of the beta-galactoside-binding lectin, galectin-3. Am J Pathol. (1996) 148:1661–70. 8623933 PMC1861566

[59] Ghiasi H Cai S Perng G-C Nesburn AB Wechsler SL. Both CD4+ and CD8+ T cells are involved in protection against HSV-1 induced corneal scarring. Br J Ophthalmol. (2000) 84:408–12. 10.1136/bjo.84.4.408 10729300 PMC1723442

[60] Gonzalez MI Rubinstein N Ilarregui JM Toscano MA Sanjuan NA Rabinovich GA. Regulated expression of galectin-1 after in vitro productive infection with herpes simplex virus type I: Implications for T cell apoptosis. Int J Immunopathol Pharmacol. (2005) 18:615–23. 10.1177/039463200501800402 16388708

[61] Johnson AJ Chu C-F Milligan GN. Effector CD4+ T cell involvement in clearance of infectious herpes simplex virus type 1 from sensory ganglia and spinal cords. J Virol. (2008) 82:9678–88. 10.1128/JVI.01159-08 18667492 PMC2546982

[62] Mikloska Z Kesson AM Penfold ME Cunningham AL. Herpes simplex virus protein targets for CD4 and CD8 lymphocyte cytotoxicity in cultured epidermal keratinocytes treated with interferon-γ. J Infect Dis. (1996) 173:7–17. 10.1093/infdis/173.1.7 8537685

[63] Ouyang J Juszczynski P Rodig SJ Green MR O'Donnell E Currie T. Viral induction and targeted inhibition of galectin-1 in EBV+ posttransplant lymphoproliferative disorders. Blood. (2011) 117:4315–22. 10.1182/blood-2010-11-320481 21300977

[64] Rajasagi NK Suryawanshi A Sehrawat S Reddy PB Mulik S Hirashima M. Galectin-1 reduces the severity of herpes simplex virus-induced ocular immunopathological lesions. J Immunol. (2012) 188:4631–43. 10.4049/jimmunol.1103063 22467659 PMC3338323

[65] Woodward AM Mauris J Argüeso P. Binding of transmembrane mucins to galectin-3 limits herpesvirus 1 infection of human corneal keratinocytes. J Virol. (2013) 87:5841–7. 10.1128/JVI.00166-13 23487460 PMC3648170

[66] Reddy PB Sehrawat S Suryawanshi A Rajasagi NK Mulik S Hirashima M. Influence of galectin-9/Tim-3 interaction on herpes simplex virus-1 latency. J Immunol. (2011) 187:5745–55. 10.4049/jimmunol.1102105 22021615 PMC3221893

[67] Freeman ML Sheridan BS Bonneau RH Hendricks RL. Psychological stress compromises CD8+ T cell control of latent herpes simplex virus type 1 infections. J Immunol. (2007) 179:322–8. 10.4049/jimmunol.179.1.322 17579052 PMC2367250

[68] Strauss G Osen W Debatin KM. Induction of apoptosis and modulation of activation and effector function in T cells by immunosuppressive drugs. Clin Exp Immunol. (2002) 128:255–66. 10.1046/j.1365-2249.2002.01777.x 11985515 PMC1906394

[69] Chernyy ES Rapoport EM Andre S Kaltner H Gabius H-J Bovin NV. Galectins promote the interaction of influenza virus with its target cell. Biochemistry. (2011) 76:958–67. 10.1134/S0006297911080128 22022970

[70] Yang M-L Chen Y-H Wang S-W Huang Y-J Leu C-H Yeh N-C. Galectin-1 binds to influenza virus and ameliorates influenza virus pathogenesis. J Virol. (2011) 85:10010–20. 10.1128/JVI.00301-11 21795357 PMC3196456

[71] Mitnaul LJ Matrosovich MN Castrucci MR Tuzikov AB Bovin NV Kobasa D. Balanced hemagglutinin and neuraminidase activities are critical for efficient replication of influenza A virus. J Virol. (2000) 74:6015–20. 10.1128/JVI.74.13.6015-6020.2000 10846083 PMC112098

[72] Chen Y Zhou J Cheng Z Yang S Chu H Fan Y. Functional variants regulating LGALS1 (Galectin 1) expression affect human susceptibility to influenza A (H7N9). Sci Rep. (2015) 5:8517. 10.1038/srep08517 25687228 PMC4649671

[73] Heslop HE. How I treat EBV lymphoproliferation. Blood. (2009) 114:4002–8. 10.1182/blood-2009-07-143545 19724053 PMC2774540

[74] Pioche-Durieu C Keryer C Souquère S Bosq J Faigle W Loew D. In nasopharyngeal carcinoma cells, Epstein-Barr virus LMP1 interacts with galectin 9 in membrane raft elements resistant to simvastatin. J Virol. (2005) 79:13326–37. 10.1128/JVI.79.21.13326-13337.2005 16227255 PMC1262583

[75] Klibi J Niki T Riedel A Pioche-Durieu C Souquere S Rubinstein E. Blood diffusion and Th1-suppressive effects of galectin-9–containing exosomes released by Epstein-Barr virus–infected nasopharyngeal carcinoma cells. Blood. (2009) 113:1957–66. 10.1182/blood-2008-02-142596 19005181

[76] Lee P-H Liu C-M Ho T-S Tsai Y-C Lin C-C Wang Y-F. Enterovirus 71 virion-associated galectin-1 facilitates viral replication and stability. PLoS ONE. (2015) 10:e0116278. 10.1371/journal.pone.0116278 25706563 PMC4338065

[77] Huang W-C Chen H-L Chen H-Y Peng K-P Lee Y Huang L-M. Galectin-3 and its genetic variation rs4644 modulate enterovirus 71 infection. PLoS ONE. (2016) 11:e0168627. 10.1371/journal.pone.0168627 28002441 PMC5176291

[78] Garner OB Aguilar HC Fulcher JA Levroney EL Harrison R Wright L. Endothelial galectin-1 binds to specific glycans on nipah virus fusion protein and inhibits maturation, mobility, and function to block syncytia formation. PLoS Pathog. (2010) 6:e1000993. 10.1371/journal.ppat.1000993 20657665 PMC2904771

[79] Garner OB Yun T Pernet O Aguilar HC Park A Bowden TA. Timing of galectin-1 exposure differentially modulates Nipah virus entry and syncytium formation in endothelial cells. J Virol. (2015) 89:2520–9. 10.1128/JVI.02435-14 25505064 PMC4325760

[80] Reyes RE González CR Jiménez RC Herrera MO Andrade AA Karunaratne DN Mechanisms of O-antigen structural variation of bacterial lipopolysaccharide (LPS). In: Karunaratne DN editor. The Complex World of Polysaccharides. Rijeka: InTech (2012). p. 71–98.

[81] Mey A Leffler H Hmama Z Normier G Revillard J-P. The animal lectin galectin-3 interacts with bacterial lipopolysaccharides via two independent sites. J Immunol. (1996) 156:1572–7. 8568262

[82] Quattroni P Li Y Lucchesi D Lucas S Hood DW Herrmann M. Galectin-3 binds *Neisseria meningitidis* and increases interaction with phagocytic cells. Cell Microbiol. (2012) 14:1657–75. 10.1111/j.1462-5822.2012.01838.x 22827322 PMC3749814

[83] Toscano MA Allo VCM Cutine AM Rabinovich GA Mariño KV. Untangling galectin-driven regulatory circuits in autoimmune inflammation. Trends Mol Med. (2018) 24:348–63. 10.1016/j.molmed.2018.02.008 29555188

[84] Mishra BB Li Q Steichen AL Binstock BJ Metzger DW Teale JM. Galectin-3 functions as an alarmin: pathogenic role for sepsis development in murine respiratory tularemia. PLoS ONE. (2013) 8:e59616. 10.1371/journal.pone.0059616 23527230 PMC3603908

[85] Díaz-Alvarez L Ortega E. The many roles of galectin-3, a multifaceted molecule, in innate immune responses against pathogens. Mediat Inflamm. (2017) 2017:9247574. 10.1155/2017/9247574 28607536 PMC5457773

[86] Li Y Komai-Koma M Gilchrist DS Hsu DK Liu F-T Springall T. Galectin-3 is a negative regulator of lipopolysaccharide-mediated inflammation. J Immunol. (2008) 181:2781–9. 10.4049/jimmunol.181.4.2781 18684969

[87] Paz I Sachse M Dupont N Mounier J Cederfur C Enninga J. Galectin-3, a marker for vacuole lysis by invasive pathogens. Cell Microbiol. (2010) 12:530–44. 10.1111/j.1462-5822.2009.01415.x 19951367

[88] Weng I-C Hsu DK Liu F-T Role of galectin-3 in macrophage response to *Listeria monocytogenes* infection (133.34). J Immunol. (2009) 182:133.34.

[89] Weng I-C Chen H-L Lo T-H Lin W-H Chen H-Y Hsu DK. Cytosolic galectin-3 and -8 regulate antibacterial autophagy through differential recognition of host glycans on damaged phagosomes. Glycobiology. (2018) 28:392–405. 10.1093/glycob/cwy017 29800364

[90] Alqahtani F Mahdavi J Wheldon LM Vassey M Pirinccioglu N Royer P-J. Deciphering the complex three-way interaction between the non-integrin laminin receptor, galectin-3 and *Neisseria meningitidis*. Open Biol. (2014) 4:140053. 10.1098/rsob.140053 25274119 PMC4221890

[91] Tamai R Kobayashi-Sakamoto M Kiyoura Y. Extracellular galectin-1 enhances adhesion to and invasion of oral epithelial cells by *Porphyromonas gingivalis*. Can J Microbiol. (2018) 64:465–71. 10.1139/cjm-2017-0461 29544077

[92] Yilmaz Ö Watanabe K Lamont RJ. Involvement of integrins in fimbriae-mediated binding and invasion by *Porphyromonas gingivalis*. Cell Microbiol. (2002) 4:305–14. 10.1046/j.1462-5822.2002.00192.x 12027958

[93] Moiseeva EP Williams B Goodall AH Samani NJ. Galectin-1 interacts with β-1 subunit of integrin. Biochem Biophys Res Commun. (2003) 310:1010–6. 10.1016/j.bbrc.2003.09.112 14550305

[94] O'Neill AM Thurston TLM Holden DW Cytosolic replication of group A *Streptococcus* in human macrophages. mBio. (2016) 7:e00020–e16. 10.1128/mBio.00020-16 27073088 PMC4959517

[95] Lu S-L Kuo C-F Chen H-W Yang Y-S Liu C-C Anderson R. Insufficient acidification of autophagosomes facilitates group a streptococcus survival and growth in endothelial cells. mBio. (2015) 6:e01435–15. 10.1128/mBio.01435-15 26419882 PMC4611045

[96] Toh H Lin C-Y Nakajima S Aikawa C Nozawa T Nakagawa I. Group A *Streptococcus* NAD-glycohydrolase inhibits caveolin 1-mediated internalization into human epithelial cells. Front Cell Infect Microbiol. (2019) 9:398. 10.3389/fcimb.2019.00398 31850237 PMC6893971

[97] Cheng Y-L Wu Y-W Kuo C-F Lu S-L Liu F-T Anderson R. Galectin-3 inhibits Galectin-8/Parkin-mediated ubiquitination of group A *streptococcus*. mBio. (2017) 8:e00899–17. 10.1128/mBio.00899-17 28743815 PMC5527311

[98] Li H Yang T Liao T Debowski AW Nilsson H-O Fulurija A. The redefinition of *Helicobacter pylori* lipopolysaccharide O-antigen and core-oligosaccharide domains. PLoS Pathog. (2017) 13:e1006280. 10.1371/journal.ppat.1006280 28306723 PMC5371381

[99] Fowler M Thomas RJ Atherton J Roberts IS High NJ Galectin-3 binds to *Helicobacter pylori* O-antigen: it is upregulated and rapidly secreted by gastric epithelial cells in response to *H. pylori* adhesion. Cell Microbiol. (2006) 8:44–54. 10.1111/j.1462-5822.2005.00599.x 16367865

[100] Subhash VV Ling SSM Ho B. Extracellular galectin-3 counteracts adhesion and exhibits chemoattraction in *Helicobacter pylori*-infected gastric cancer cells. Microbiology. (2016) 162:1360–6. 10.1099/mic.0.000322 27283429

[101] Park A-M Hagiwara S Hsu DK Liu F-T Yoshie O. Galectin-3 plays an important role in innate immunity to gastric infection by *Helicobacter pylori*. Infect Immun. (2016) 84:1184–93. 10.1128/IAI.01299-15 26857579 PMC4807496

[102] Yong X Tang B Li B-S Xie R Hu C-J Luo G. *Helicobacter pylori* virulence factor CagA promotes tumorigenesis of gastric cancer via multiple signaling pathways. Cell Commun Signal. (2015) 13:30. 10.1186/s12964-015-0111-0 26160167 PMC4702319

[103] Farhad M Rolig AS Redmond WL. The role of Galectin-3 in modulating tumor growth and immunosuppression within the tumor microenvironment. Oncoimmunology. (2018) 7:e1434467. 10.1080/2162402X.2018.1434467 29872573 PMC5980349

[104] Díaz P Valenzuela Valderrama M Bravo J Quest AFG. *Helicobacter pylori* and gastric cancer: adaptive cellular mechanisms involved in disease progression. Front Microbiol. (2018) 9:5. 10.3389/fmicb.2018.00005 29403459 PMC5786524

[105] Yang C Deng S High level of *Helicobacter pylori* infection contributes to the survival of colon cancer via up-regulating autophagy *in vitro*. Int J Clin Exp Med. (2018) 11:11901–10.

[106] Li F-Y Weng I-C Lin C-H Kao M-C Wu M-S Chen H-Y. *Helicobacter pylori* induces intracellular galectin-8 aggregation around damaged lysosomes within gastric epithelial cells in a host O-glycan-dependent manner. Glycobiology. (2018) 29:151–62. 10.1093/glycob/cwy095 30289459

[107] Chen W-S Cao Z Truong L Sugaya S Panjwani N. Fingerprinting of galectins in normal, *P. aeruginosa*–infected, and chemically burned mouse corneas. Invest Ophthalmol Vis Sci. (2015) 56:515–25. 10.1167/iovs.14-15338 25564452 PMC4303041

[108] Gupta SK Masinick S Garrett M Hazlett LD. *Pseudomonas aeruginosa* lipopolysaccharide binds galectin-3 and other human corneal epithelial proteins. Infect Immun. (1997) 65:2747–53. 10.1128/IAI.65.7.2747-2753.1997 9199445 PMC175387

[109] Ye P Garvey PB Zhang P Nelson S Bagby G Summer WR. Interleukin-17 and lung host defense against *Klebsiella pneumoniae* infection. Am J Respir Cell Mol Biol. (2001) 25:335–40. 10.1165/ajrcmb.25.3.4424 11588011

[110] Wang F Xu J Liao Y Wang Y Liu C Zhu X. Tim-3 ligand galectin-9 reduces IL-17 level and accelerates *Klebsiella pneumoniae* infection. Cell Immunol. (2011) 269:22–8. 10.1016/j.cellimm.2011.03.005 21453908

[111] Tolson DL Harrison BA Latta RK Lee KK Altman E. The expression of nonagglutinating fimbriae and its role in Proteus mirabilis adherence to epithelial cells. Can J Microbiol. (1997) 43:709–17. 10.1139/m97-102 9304781

[112] Altman E Harrison BA Latta RK Lee KK Kelly JF Thibault P. Galectin-3-mediated adherence of *Proteus mirabilis* to Madin-Darby canine kidney cells. Biochem Cell Biol. (2001) 79:783–8. 10.1139/o01-135 11800020

[113] Lee KK Harrison BA Latta R Altman E. The binding of *Proteus mirabilis* nonagglutinating fimbriae to ganglio-series asialoglycolipids and lactosyl ceramide. Can J Microbiol. (2000) 46:961–6. 10.1139/w00-083 11068685

[114] Davicino RC Méndez-Huergo SP Eliçabe RJ Stupirski JC Autenrieth I Di Genaro MS. Galectin-1–driven tolerogenic programs aggravate *Yersinia enterocolitica* infection by repressing antibacterial immunity. J Immunol. (2017) 199:1382–92. 10.4049/jimmunol.1700579 28716827

[115] Grabowski B Schmidt MA Rüter C. Immunomodulatory *Yersinia* outer proteins (Yops)–useful tools for bacteria and humans alike. Virulence. (2017) 8:1124–47. 10.1080/21505594.2017.1303588 28296562 PMC5711447

[116] Campbell LA Lee A Kuo C. Cleavage of the N-linked oligosaccharide from the surfaces of chlamydia species affects infectivity in the mouse model of lung infection. Infect Immun. (2006) 74:3027–9. 10.1128/IAI.74.5.3027-3029.2006 16622244 PMC1459694

[117] Lujan AL Croci DO Tudela JAG Losinno AD Cagnoni AJ Mariño KV. Glycosylation-dependent galectin–receptor interactions promote *Chlamydia trachomatis* infection. Proc Natl Acad Sci USA. (2018) 115:E6000–9. 10.1073/pnas.1802188115 29891717 PMC6042088

[118] Pineda MA Corvo L Soto M Fresno M Bonay P. Interactions of human galectins with *Trypanosoma cruzi*: binding profile correlate with genetic clustering of lineages. Glycobiology. (2014) 25:197–210. 10.1093/glycob/cwu103 25267603

[119] Freire-de-Lima L Oliveira IA Neves JL Penha LL Alisson-Silva F Dias WB. Sialic acid: a sweet swing between mammalian host and Trypanosoma cruzi. Front Immunol. (2012) 3:356. 10.3389/fimmu.2012.00356 23230438 PMC3515882

[120] Esch KJ Petersen CA. Transmission and epidemiology of zoonotic protozoal diseases of companion animals. Clin Microbiol Rev. (2013) 26:58–85. 10.1128/CMR.00067-12 23297259 PMC3553666

[121] Kleshchenko YY Moody TN Furtak VA Ochieng J Lima MF Villalta F. Human galectin-3 promotes *Trypanosoma cruzi* adhesion to human coronary artery smooth muscle cells. Infect Immun. (2004) 72:6717–21. 10.1128/IAI.72.11.6717-6721.2004 15501810 PMC523038

[122] Moody TN Ochieng J Villalta F. Novel mechanism that T*rypanosoma cruzi* uses to adhere to the extracellular matrix mediated by human galectin-3. FEBS Lett. (2000) 470:305–8. 10.1016/S0014-5793(00)01347-8 10745086

[123] Marchiori MF Riul TB Bortot LO Andrade P Junqueira GG Foca G. Binding of triazole-linked galactosyl arylsulfonamides to galectin-3 affects *Trypanosoma cruzi* cell invasion. Bioorg Med Chem. (2017) 25:6049–59. 10.1016/j.bmc.2017.09.042 29032929

[124] Benatar AF García GA Bua J Cerliani JP Postan M Tasso LM. Galectin-1 prevents infection and damage induced by *Trypanosoma cruzi* on cardiac cells. PLoS Negl Trop Dis. (2015) 9:e0004148. 10.1371/journal.pntd.0004148 26451839 PMC4599936

[125] Ferrer MF Pascuale CA Gomez RM Leguizamon MS. DTU I isolates of *Trypanosoma cruzi* induce upregulation of Galectin-3 in murine myocarditis and fibrosis. Parasitology. (2014) 141:849–58. 10.1017/S0031182013002254 24533969

[126] da Silva AA Teixeira TL Teixeira SC Machado FC dos Santos MA Tomiosso TC. Galectin-3: A friend but not a foe during *Trypanosoma cruzi* experimental infection. Front Cell Infect Microbiol. (2017) 7:463. 10.3389/fcimb.2017.00463 29164071 PMC5675870

[127] Pineda MA Cuervo H Fresno M Soto M Bonay P. Lack of galectin-3 prevents cardiac fibrosis and effective immune responses in a murine model of *Trypanosoma cruzi* infection. J Infect Dis. (2015) 212:1160–71. 10.1093/infdis/jiv185 25805753

[128] Singh BN Lucas JJ Beach DH Shin ST Gilbert RO. Adhesion of *Tritrichomonas foetus* to bovine vaginal epithelial cells. Infect Immun. (1999) 67:3847–54. 10.1128/IAI.67.8.3847-3854.1999 10417148 PMC96664

[129] Singh BN Hayes GR Lucas JJ Sommer U Viseux N Mirgorodskaya E. Structural details and composition of *Trichomonas vaginalis* lipophosphoglycan in relevance to the epithelial immune function. Glycoconj J. (2009) 26:3–17. 10.1007/s10719-008-9157-1 18604640 PMC2637367

[130] Bastida-Corcuera FD Okumura CY Colocoussi A Johnson PJ. *Trichomonas vaginalis* lipophosphoglycan mutants have reduced adherence and cytotoxicity to human ectocervical cells. Eukaryot Cell. (2005) 4:1951–8. 10.1128/EC.4.11.1951-1958.2005 16278462 PMC1287856

[131] Okumura CYM Baum LG Johnson PJ. Galectin-1 on cervical epithelial cells is a receptor for the sexually transmitted human parasite *Trichomonas vaginalis*. Cell Microbiol. (2008) 10:2078–90. 10.1111/j.1462-5822.2008.01190.x 18637021 PMC4437540

[132] Fichorova RN Yamamoto HS Fashemi T Foley E Ryan S Beatty N. *Trichomonas vaginalis* lipophosphoglycan exploits binding to galectin-1 and-3 to modulate epithelial immunity. J Biol Chem. (2016) 291:998–1013. 10.1074/jbc.M115.651497 26589797 PMC4705417

[133] Oakley MS Majam V Mahajan B Gerald N Anantharaman V Ward JM. Pathogenic roles of CD14, galectin-3, and OX40 during experimental cerebral malaria in mice. PLoS ONE. (2009) 4:e6793. 10.1371/journal.pone.0006793 19710907 PMC2728507

[134] Toscano MA Tongren JE Souza JBD Liu F-T Riley EM Rabinovich GA. Endogenous galectin-3 controls experimental malaria in a species-specific manner. Parasite Immunol. (2012) 34:383–7. 10.1111/j.1365-3024.2012.01366.x 22486577

[135] Bunn PT de Montes Oca M de Rivera FL Kumar R Edwards CL Faleiro RJ. Galectin-1 impairs the generation of anti-parasitic Th1 cell responses in the liver during experimental visceral *Leishmaniasis. Front*. Immunol. (2017) 8:1307. 10.3389/fimmu.2017.01307 29075269 PMC5643427

[136] Hatanaka O Rezende CP Moreno P Freitas Fernandes F Oliveira Brito PKM Martinez R. Galectin-3 inhibits paracoccidioides brasiliensis growth and impacts paracoccidioidomycosis through multiple mechanisms. mSphere. (2019) 4:e00209–19. 10.1128/mSphere.00209-19 31019001 PMC6483048

[137] Jouault T El Abed-El Behi M Martínez-Esparza M Breuilh L Trinel P-A Chamaillard M. Specific recognition of *Candida albicans* by macrophages requires galectin-3 to discriminate *Saccharomyces cerevisiae* and needs association with TLR2 for signaling. J Immunol. (2006) 177:4679–87. 10.4049/jimmunol.177.7.4679 16982907

[138] Linden JR Kunkel D Laforce-Nesbitt SS Bliss JM. The role of galectin-3 in phagocytosis of *Candida albicans* and *Candida parapsilosis* by human neutrophils. Cell Microbiol. (2013) 15:1127–42. 10.1111/cmi.12103 23279221 PMC3640745

[139] Wu S-Y Huang J-H Chen W-Y Chan Y-C Lin C-H Chen Y-C. Cell intrinsic galectin-3 attenuates neutrophil ROS-dependent killing of *Candida* by modulating CR3 downstream Syk activation. Front Immunol. (2017) 8:48. 10.3389/fimmu.2017.00048 28217127 PMC5289966

[140] Linden JR De Paepe ME Laforce-Nesbitt SS Bliss JM. Galectin-3 plays an important role in protection against disseminated candidiasis. Med Mycol. (2013) 51:641–51. 10.3109/13693786.2013.770607 23488971 PMC3713172

[141] Woods JP. Knocking on the right door and making a comfortable home: *Histoplasma capsulatum* intracellular pathogenesis. Curr Opin Microbiol. (2003) 6:327–31. 10.1016/S1369-5274(03)00080-8 12941399

[142] Kroetz DN Deepe GS. The role of cytokines and chemokines in *Histoplasma capsulatum* infection. Cytokine. (2012) 58:112–7. 10.1016/j.cyto.2011.07.430 21871816 PMC3227768

[143] Cooper DN Boulianne RP Charlton S Farrell EM Sucher A Lu BC. Fungal galectins, sequence and specificity of two isolectins from *Coprinus cinereus*. J Biol Chem. (1997) 272:1514–21. 10.1074/jbc.272.3.1514 8999822

[144] Karakostis K Costa C Zito F Matranga V. Heterologous expression of newly identified galectin-8 from sea urchin embryos produces recombinant protein with lactose binding specificity and anti-adhesive activity. Sci Rep. (2015) 6:19169. 10.1038/srep19169 26640155 PMC4671058

[145] Vasta GR Ahmed H Du S-J Henrikson D. Galectins in teleost fish: Zebrafish (*Danio rerio*) as a model species to address their biological roles in development and innate immunity. Glycoconj J. (2004) 21:503–21. 10.1007/s10719-004-5541-7 15750792

[146] Kamhawi S Ramalho-Ortigao M Pham VM Kumar S Lawyer PG Turco SJ. A role for insect galectins in parasite survival. Cell. (2004) 119:329–41. 10.1016/j.cell.2004.10.009 15543683

[147] Shi W Xue C Su X Lu F. The roles of galectins in parasitic infections. Acta Trop. (2018) 177:97–104. 10.1016/j.actatropica.2017.09.027 28986248 PMC5672833

[148] Jones S Parasitology: Stuck in the gut. Nat Rev Microbiol. (2005) 3:5 10.1038/nrmicro1079

[149] Lu M Tian X Yang X Yuan C Ehsan M Liu X The N- and C-terminal carbohydrate recognition domains of *Haemonchus contortus* galectin bind to distinct receptors of goat PBMC and contribute differently to its immunomodulatory functions in host-parasite interactions. Parasit Vectors. (2017) 10:409 10.1186/s13071-017-2353-8 28870237 PMC5584048

[150] Xu J Yang F Jiang P Liu RD Zhang X Cui J. Molecular characterization of *Trichinella spiralis* galectin and its participation in larval invasion of host's intestinal epithelial cells. Vet Res. (2018) 49:79. 10.1186/s13567-018-0573-3 30068382 PMC6071371

[151] González-Miguel J Morchón R Siles-Lucas M Oleaga A Simón F. Surface-displayed glyceraldehyde 3-phosphate dehydrogenase and galectin from Dirofilaria immitis enhance the activation of the fibrinolytic system of the host. Acta Trop. (2015) 145:8–16. 10.1016/j.actatropica.2015.01.010 25666684

[152] González-Miguel J Larrazabal C Loa-Mesón D Siles-Lucas M Simón F Morchón R. Glyceraldehyde 3-phosphate dehydrogenase and galectin from *Dirofilaria immitis* participate in heartworm disease endarteritis via plasminogen/plasmin system. Vet Parasitol. (2016) 223:96–101. 10.1016/j.vetpar.2016.04.029 27198784

[153] Peng G Sun D Rajashankar KR Qian Z Holmes KV Li F. Crystal structure of mouse coronavirus receptor-binding domain complexed with its murine receptor. Proc Natl Acad Sci USA. (2011) 108:10696–701. 10.1073/pnas.1104306108 21670291 PMC3127895

[154] Tortorici MA Walls AC Lang Y Wang C Li Z Koerhuis D. Structural basis for human coronavirus attachment to sialic acid receptors. Nat Struct Mol Biol. (2019) 26:481–9. 10.1038/s41594-019-0233-y 31160783 PMC6554059

[155] Suzuki O Abe M Hashimoto Y. Sialylation by β-galactoside α-2, 6-sialyltransferase and N-glycans regulate cell adhesion and invasion in human anaplastic large cell lymphoma. Int J Oncol. (2015) 46:973–80. 10.3892/ijo.2015.2818 25573487 PMC4324587

[156] Lv R Bao Q Li Y. Regulation of M1-type and M2-type macrophage polarization in RAW264. 7 cells by Galectin-9. Mol Med Rep. (2017) 16:9111–9. 10.3892/mmr.2017.7719 28990062

[157] MacKinnon AC Farnworth SL Hodkinson PS Henderson NC Atkinson KM Leffler H. Regulation of alternative macrophage activation by galectin-3. J Immunol. (2008) 180:2650–8. 10.4049/jimmunol.180.4.2650 18250477

[158] Novak R Dabelic S Dumic J. Galectin-1 and galectin-3 expression profiles in classically and alternatively activated human macrophages. Biochim Biophys Acta. (2012) 1820:1383–90. 10.1016/j.bbagen.2011.11.014 22155450

[159] Gittens BR Bodkin JV Nourshargh S Perretti M Cooper D. Galectin-3: a positive regulator of leukocyte recruitment in the inflamed microcirculation. J Immunol. (2017) 198:4458–69. 10.4049/jimmunol.1600709 28438899 PMC5444525

[160] Klyosov AA Traber PG Galectins in disease and potential therapeutic approaches. In: Galectins and Disease Implications for Targeted Therapeutics. ACS Symposium Series; American Chemical Society (2012). p. 3–43.

