# Supplementary material for: Utilization of Galectins by Pathogens for Infection
Source: Front Immunol. 2020 Aug 19;11:1877. doi: 10.3389/fimmu.2020.01877 (PMC7466766; doi:10.3389/fimmu.2020.01877)
Supplement: Supplementary file 1 [file Table_1.docx]

| **Pathogen** | **Host cell type(s) involved in galectin targeting** | **Galectin involved** | **Host/pathogen ligands involved in galectin mediated interaction** | | **Associated function facilitating infection** | **References** |
| --- | --- | --- | --- | --- | --- | --- |
|  |  |  | **Pathogen ligand** | **Host ligand** |  |  |
| **Viruses** | | | | | | |
| HIV-1 | T cells | Gal-1 | Complex glycans with 2 to 6 β-galactoses | CD 4+ T cell receptor | Enhances adherence to host cells | St-Pierre et al. (2011);  Mercier et al. (2008) |
|  |  | Gal-3 | Gag-P6 viral protein | ALG-2-interacting protein X (Alix) | Enhances HIV-1 budding and cell-cell viral transmission | Wang et al. (2014) |
|  |  | Gal-9 | - | Protein disulfide isomerase (PDI) | Enhances membrane fusion, viral entry | Bi et al. (2008) |
|  |  |  | - | Tim-3 | Induces apoptosis of Th1 cells |  |
| HTLV-1 | T cells | Gal-1 | *Viral *N*-glycans on gp46 | - | Enhances virological synapse formation and adherence to host cells | Gauthier et al. (2008);  Arp et al. (19996) |
|  |  | Gal-3 | - | - | *Involved in cell-to-cell transmission of virus | Pais-Correia et al. (2010);  Hsu et al. (1996) |
| HSV-1 | T cell subtypes including Th1, Th17 and cytotoxic T cells | Gal-1 | - | Cell surface *N*- or *O*-glycans with LacNAc | Induces apoptosis of T cells | Gonzalez et al. (2005) |
|  | Corneal keratinocytes | Gal-3 | - | - | Enhances adherence to host cells | Woodward et al. (2013) |
|  | CD8+ T cell accumulated in the trigeminal ganglion | Gal-9 | - | Tim-3 | Enhances virus reactivation from latency either by decreasing cell count or by increasing the effector function of CD8+ T cells | Reddy et al. (2011) |
| Influenza | Alveolar epithelial cells | Gal-1 and Gal-8 | Galactose residues | - | Enhances adherence to host cells | Chernyy et al. (2011) |
| EBV | CD8+ T cells | Gal-1 | - | Cell surface *N*- or *O*-glycans with LacNAc | Induces apoptosis of CD8+ T cells | Ouyang et al. (2011) |
|  | CD4+ T cells | Gal-9 | - | Tim-3 | Induces apoptosis of CD4+ T cells | Klibi et al. (2009) |
| EV71 | Targets cells in different tissues | Gal-1 | Glycosylated capsid proteins | - | Enhances viral replication, virion release and resistance to environment stress | Lee et al. (2015) |
|  |  | Gal-3 | - | - | Enhances virion release | Huang et al. (2016) |
| Nipha virus | Endothelial cells | Gal-1 | Complex *N*-glycans of NiV-F and Niv-G envelope proteins | Host cell surface complex *N*-glycans | Enhances adherence to host cells | Garner et al. (2010);  Garner et al. (2015) |
| **Bacteria** | | | | | | |
| *Salmonella spp.* | Macrophages | Gal-3 | LPS | - | Reduces endotoxic shock and decreases bactericidal NO production | Li et al. (2008) |
| *L. monocytogenes* | Macrophages | Gal-3 | - | - | Decreases bactericidal NO production | Weng et al. (2009) |
|  |  |  | - | - | Interferes with autophagy mediated killing of bacteria | Weng et al. (2018) |
| *N. meningitidis* | Phagocytic cells: Macrophages, monocytes | Gal-3 | LPS immunotypes with terminal LacNAc | - | Enhances adherence to host cells | Quattroni et al. (2012) |
|  |  |  | Bacterial Pili, secretin | Non-integrin laminin receptor | Strengthens initial contact to host cells | Alqahtani et al. (2014) |
| *P. gingivalis* | Gingival epithelial cells | Gal-1 | Microbial glycans: O-antigen polysaccharide in LPS | β1-integrin oligosaccharide chains | Enhances adherence to host cells | Tamai et al. (2018) |
|  | Macrophages |  | - | *Lipid rafts and integrins | *Contributes to lipid raft mediated intracellular uptake | Moiseeva et al. (2003); Tamai et al., (2018) |
| Group A *Streptococcus* | Endothelial cells | Gal-3 | - | - | Interferes with autophagy mediated killing of bacteria | Cheng et al. (2017) |
| *H. pylori* | Gastric epithelial cells | Gal-3 | O-antigen side chain with LacNAc | *Glycans on basement membrane | Enhances adherence to host cells and colonization | Fowler et al. (2006) |
|  |  | Gal-8 | - | Vacuolar *O*-glycans exposed from damaged lysosomes | * Contributes to form autophagy vesicles | Li et al. (2018); Yang and Deng, (2018) |
| *P. aeruginosa* | Corneal epithelial cells | Gal-3 | LPS outer core | - | Enhances adherence to host cells | Gupta et al. (1997) |
| *K. pneumoniae* | Th1 and Th17 cells | Gal-9 | - | *Tim-3 | Induces apoptosis of Th1 and Th17 cells and inhibits neutrophil differentiation | Wang et al. (2011) |
| *P. mirabilis* | Uroepithelial cells | Gal-3 | Non-agglutinating fimbriae | - | Enhances adherence to host cells | Altman et al. (2001) |
| *Y. enterocolitica* | Spleen and Peyer’s patches | Gal-1 | *Yersinia* outer proteins | - | Mitigates mucosal proinflammatory responses | Davicino et al. (2017) |
| *C. trachomatis* | Cervical epithelial cells | Gal-1 | Glycoproteins with terminal LacNAc | PDGFRβ and β1/αVβ3 integrins, complex β1,6 branched N-glycans | Enhances adhesion and internalization to host cells | Lujn et al. (2018) |
| **Parasites** | | | | | | |
| *T. cruzi* | Different cells vulnerable to infection | Gal-3 | *Surface glycoproteins of trypanosome | - | Enhances adherence to host cells and to laminin | Moody et al. (2000);  Kleshchenko et al. (2004) |
|  |  | Gal-7 | - | Glycans on epimastigotes | *Enhances adherence to host cells | Pineda et al. (2014) |
|  |  | Gal-8 | - | Glycans on trypomastigoes | Enhances adhesion to host cells |  |
| *T. vaginalis* | Vaginal epithelial cells | Gal-1 | Lipophosphogly-cans | - | Enhances adherence to host cells and subverts the immune responses | Okumura et al. (2008); Fichorova et al. 2016 |
| *Plasmodium spp.* | Red blood cells | Gal-3 | - | - | *Modulates anti-IgG against *P. yoelii* merozoite surface protein 119 | Oakley et al. (2009); Toscano et al. (2012) |
| *L. donovani* | Th1 and Tr1 cells | Gal-1 | - | - | *Reduces anti-parasitic immune responses by apoptosis of T cells | Bunn et al. (2017) |
| **Fungi** | | | | | | |
| *C. albicans* | Neutrophils | Gal-3 | - | Syk tyrosine kinase | Suppresses reactive oxygen species production | Wu et al. (2017) |
| *H. capsulatum* | Neutrophils and dendritic cells | Gal-3 | - | - | Reduces production of Th17 cytokines | Wu et al. (2013) |

**Table 1: Galectin mediated host-pathogen interactions in favor of infection (- information not found, *assumptions)**
